# Climate transition at the Eocene–Oligocene influenced by bathymetric changes to the Atlantic–Arctic oceanic gateways

**DOI:** 10.1073/pnas.2115346119

**Published:** 2022-04-21

**Authors:** Eivind O. Straume, Aleksi Nummelin, Carmen Gaina, Kerim H. Nisancioglu

**Affiliations:** ^a^Centre for Earth Evolution and Dynamics, Department of Geosciences, University of Oslo, Oslo, 0371 Norway;; ^b^The Jackson School of Geosciences, University of Texas at Austin, Austin, TX 78712;; ^c^NORCE Norwegian Research Centre AS, Bjerknes Centre for Climate Research, Bergen, 5838 Norway;; ^d^School of Earth and Atmospheric Sciences, Queensland University of Technology, Brisbane, QLD, 4072 Australia;; ^e^Department of Earth Science, University of Bergen, Bergen, 7803 Norway

**Keywords:** paleoclimate, paleogeography, oceanic gateways, tectonics, ocean circulation

## Abstract

The Eocene–Oligocene Transition (∼33.9 Ma) marks the largest step transformation within the Cenozoic cooling trend and is characterized by a sudden growth of the Antarctic ice sheets, cooling of the interior ocean, and the establishment of strong meridional temperature gradients. Here we examine the climatic impact of oceanic gateway changes at the Eocene–Oligocene Transition by implementing detailed paleogeographic reconstructions with realistic paleobathymetric models for the Atlantic–Arctic basins in a state-of-the-art earth system model (the Norwegian Earth System Model [NorESM-F]). We demonstrate that the warm Eocene climate is highly sensitive to depth variations of the Greenland–Scotland Ridge and the proto–Fram Strait as they control the freshwater leakage from the Arctic to the North Atlantic. Our results, and proxy evidence, suggest that changes in these gateways controlled the ocean circulation and played a critical role in the growth of land-based ice sheets, alongside CO_2_-driven global cooling. Specifically, we suggest that a shallow connection between the Arctic and North Atlantic restricted the southward flow of fresh surface waters during the Late Eocene allowing for a North Atlantic overturning circulation. Consequently, the Southern Hemisphere cooled by several degrees paving the way for the glaciation of Antarctica. Shortly after, the connection to the Arctic deepened due to weakening dynamic support from the Iceland Mantle Plume. This weakened the North Atlantic overturning and cooled the Northern Hemisphere, thereby promoting glaciations there. Our study points to a controlling role of the Northeast Atlantic gateways and decreasing atmospheric CO_2_ in the onset of glaciations in both hemispheres.

## Significance

The results show that dynamic variations in the Earth’s interior could have played a key role in the Eocene–Oligocene climatic transition (∼33.9 Ma) and the inception of glaciations. Pulsations in the Iceland mantle plume modified the bathymetry of the Greenland–Scotland Ridge, which affected deep water formation in the North Atlantic. Our model simulations show that the changes in the Atlantic–Arctic oceanic gateways cooled the Southern Hemisphere, and later the Northern Hemisphere, paving the way for the growth of major land-based ice sheets. This supplements the current view that decreasing atmospheric CO_2_ concentrations and/or changes to the Southern Ocean gateways or the Tethys Seaway dominated climate changes and the inception of glaciations at the time.

The Eocene–Oligocene Transition (EOT; ∼33.9 Ma) occurred in less than 400 kyr and marks the onset of major Antarctic glaciations (e.g., refs. 1–5). Although the sharp transition was followed by warmer periods in the Oligocene and the Mid-Miocene, there have been permanent ice sheets on Antarctica ever since (e.g., ref. 6). Today’s climate, with steep pole to equator temperature gradients and polar ice caps, is rooted in events that took place at the EOT (7). In the marine δ^18^O record, the EOT is represented by a distinct positive anomaly Oi-1, which is accompanied by a positive excursion in mean δ^13^C oceanic dissolved inorganic carbon and increased biogenic sedimentation rates (1). The transition is also associated with a deepening of the ocean calcite compensation depth (2), a distinct northward migration of the Intertropical Convergence Zone (ITCZ) (8), high-latitude cooling (9), and increased Northern Hemisphere seasonality (10). It is argued that the δ^18^O anomaly observed across the EOT is too large to be explained solely by the Antarctic glaciation, and it therefore must have been accompanied by either a global cooling event or contemporaneous Northern Hemisphere glaciations (2). However, evidence for Northern Hemisphere glaciations is sparse, except for records of continentally derived ice rafted debris on the East Greenland margin (11).

There are several proposed trigger mechanisms for the EOT climatic event: 1) decreasing atmospheric CO_2_ concentrations through the Eocene forcing the Antarctic glaciations (e.g., refs. 5, 7), 2) a combination of decreasing CO_2_ and a favorable orbital configuration (2), 3) the initiation of the Antarctic Circumpolar Current (ACC) due to tectonic opening of the Southern Ocean gateways (i.e., the Drake Passage and the Tasman Gateway) (3), and 4) tectonic changes in either the Atlantic–Arctic oceanic gateways or the Tethys Seaway initiating a precursor to the Atlantic Meridional Overturning Circulation (AMOC) (12–15). Plate tectonic–related changes like mechanisms 3 and 4 could also have triggered variations in atmospheric CO_2_, either by increasing silicate weathering due to, for example, the uplift associated with the Himalayan orogen (16) or enhanced weathering and CO_2_ drawdown caused by increased precipitation on land due to a strengthening of the AMOC (17).

Paleogeography is one of the most important boundary conditions in modeling deep-time paleoclimate (e.g., refs. 15, 18). However, until recently, global paleogeographic reconstructions capturing the detailed evolution of both the Northern and Southern Hemisphere Cenozoic oceanic gateways have been lacking. In particular, the opening of the Northeast Atlantic Ocean and the subsidence history of the Greenland–Scotland Ridge (GSR) have mostly been neglected. From Late Eocene to Early Oligocene, the Southern Ocean gateways deepened, the Tethys Seaway shallowed (e.g., refs. 19, 20), and the GSR depths fluctuated due to variations in the Iceland plume activity (21–23).

In the Early Eocene, the Atlantic–Arctic connection is considered to be closed. The Northeast Atlantic Ocean started to open at ca. 54 Ma (e.g., refs. 24, 25), but its northern part (the Nordic Seas) was disconnected from the rest of the North Atlantic for most of the Eocene, being blocked by a shallow/subaerial GSR (26–28). Starting in the Middle to Late Eocene and into the Late Eocene, parts of the GSR submerged but stayed shallow (∼100 to 200 m below sea level), and in the Late Eocene it uplifted again due to increased dynamic support from the Iceland mantle plume (23). From the EOT and into the Early Oligocene, the ridge subsided, and the Faroe–Shetland Channel deepened (*SI Appendix*, Fig. S1). After this time, uplift and subsidence of the GSR, related to temporal pulsations in the Iceland Plume, have been proposed to control the production of Northern Component Water, a precursor to the modern North Atlantic Deep Water (29–31).

**Fig. 1.**
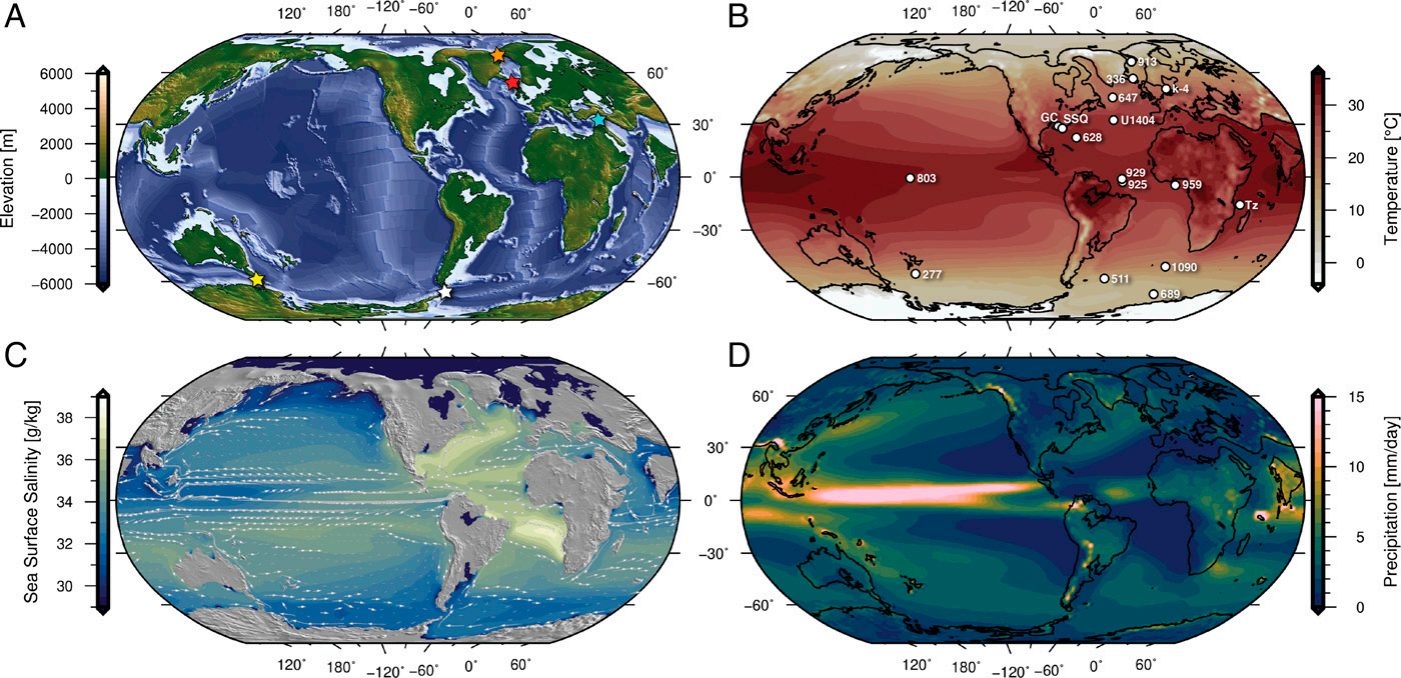
Global models at the Eocene–Oligocene Transition (ca. 34 Ma). (*A*) Reconstructed paleogeography. Colored stars mark the locations of the oceanic gateways investigated in this study. Orange star indicates Fram Strait, red star indicates GSR, cyan star indicates Tethys Seaway, yellow star indicates Tasman Gateway, and white star indicates Drake Passage. (*B*) NorESM Late Eocene simulation surface air temperature. White circles mark the paleolocation of the proxies shown in Fig. 2 and/or referred to in the main text. k-4, Kyssing-4 drill site (32), North Sea; GC, Gulf Coast samples (33); SSQ, St. Stephens Quarry (34); and Tz, Tanzania drill sites (35, 36). Data from the remaining drill sites are from the compilations of refs. 9 and 37. (*C*) Sea surface salinity. Ocean current trajectories at 50 m depth are superimposed to show the trends in surface circulation. (*D*) Mean daily precipitation.

Here we make use of the global paleogeography model of Straume et al. (23) including the paleobathymetric evolution of the oceanic gateways described above. We have developed Late Eocene (34 Ma) reconstructions (Fig. 1*A*), including realistic maximum/minimum depth scenarios of the GSR, Fram Strait, Tethys Seaway, and Southern Ocean gateways (Table 1). The different reconstructions are implemented in the Norwegian Earth System Model (NorESM-F) (38), which we use to examine the influence of major gateway changes on the Eocene–Oligocene climate transition.

**Table 1.** Overview oceanic gateway configurations for the NorESM sensitivity experiments

	Gateways				
	Fram Strait	GSR	Tethys	Drake	Tasman
Shallow/deep	20 m/140 m	70 m/660 m	150 m/770 m	490 m/990 m	330 m/1,080 m
Grid cells changed	2	8	18	16	33
Late Eocene	Deep	Shallow	Deep	Shallow	Shallow
Case 1	Deep	Shallow	Shallow	Deep	Deep
Case 2	Deep	Deep	Deep	Shallow	Shallow
Case 3	Deep	Deep	Deep	Deep	Deep
Case 4	Deep	Deep	Shallow	Deep	Deep
Case 5	Shallow	Deep	Deep	Shallow	Shallow

The shallow/deep scenarios are realistic reconstructions representing the minimum/maximum sill depth of the different oceanic gateways at 34 Ma.

## Results

**Fig. 2.**
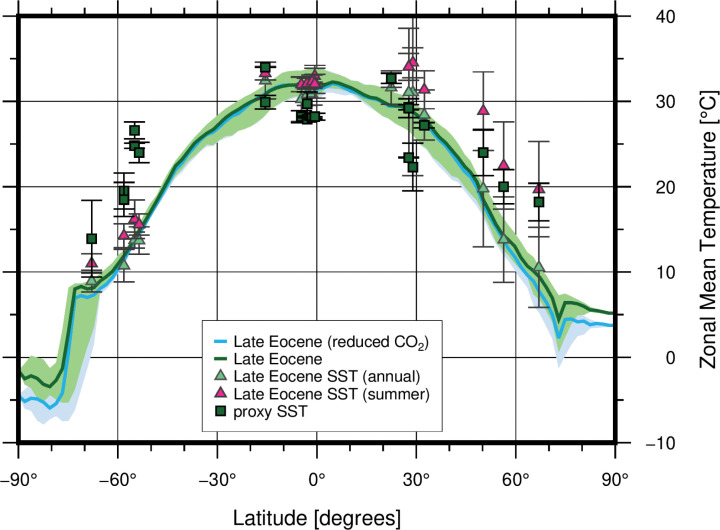
Simulated zonal mean air temperatures and SSTs, compared to SST proxies. The green line represents the simulated zonal mean surface air temperatures from the Late Eocene simulation. The blue line is the reduced CO_2_ case (Late Eocene, reduced CO_2_), with the same paleogeographic configuration as the Late Eocene. The light green and light blue shaded regions show the 25 to 75% range in the annual mean temperatures (model year 2650 to 2700). The green squares with error bars are SST proxy compilation from (37). The geographical locations of the proxies are shown in Fig. 1*B*. Light green triangles are the annual mean SST at the proxy locations, while the pink triangles are the summer SSTs at the proxy locations. Zonal mean temperatures of more cases are shown in *SI Appendix*, Fig. S4.

The Late Eocene simulation (Fig. 1) is a likely paleogeographic scenario for ∼34 Ma where the GSR is subaerial (the shallow case in Table 1), the Southern Ocean gateways are shallow, and the Tethys Seaway is open (see Table 1 for the gateway configurations and *Materials and Methods* for detailed model descriptions). The model setup with this oceanic gateway configuration sustains a proto-AMOC and a warm Northern Hemisphere, as well as a warm Antarctic continent (Figs. 1 and 2). The proto-AMOC is driven by deep water formation in the Labrador Sea and the Irminger Sea. There is no ACC in this simulation (see Fig. 4*C*), and the (proto)–Southern Ocean is dominated by subpolar gyres in the Atlantic and Pacific sectors which transport warm water from lower latitudes toward Antarctica keeping the continent warm. The midlatitude ocean circulation is characterized by gyre circulation with western boundary currents at their rims (Fig. 1*C* and *SI Appendix*, Fig. S5). While the circulation is broadly similar to today, some differences exist: For example, the Agulhas retroflection where part of the Agulhas current turns eastward sits north of the southern tip of Africa (which is located farther south than today)—a somewhat similar configuration to what has also been suggested for the glacial periods (39). In the tropical Pacific, dominant features of the surface circulation are the southern and northern equatorial currents that transport water westward. Since Australia lies farther to the south than today, the southern equatorial current connects the Pacific and the Indian Oceans ending at the eastern coast of Africa. Because the Central American Seaway is open, the North Brazil Current connects the Tropical Atlantic to the Pacific. At the subsurface (core around 150 m depth), the equatorial undercurrent flows eastward throughout the tropics from the eastern coast of Africa through the Indian, the Pacific, and the Atlantic Oceans and ends at the western coast of Africa. Altogether, the tropical ocean circulation is interconnected across the ocean basins, unlike the present as continents block the circulation. The simulated sea surface temperatures (SSTs) compare well with Late Eocene surface ocean temperature proxies (37) (Fig. 2).

**Fig. 3.**
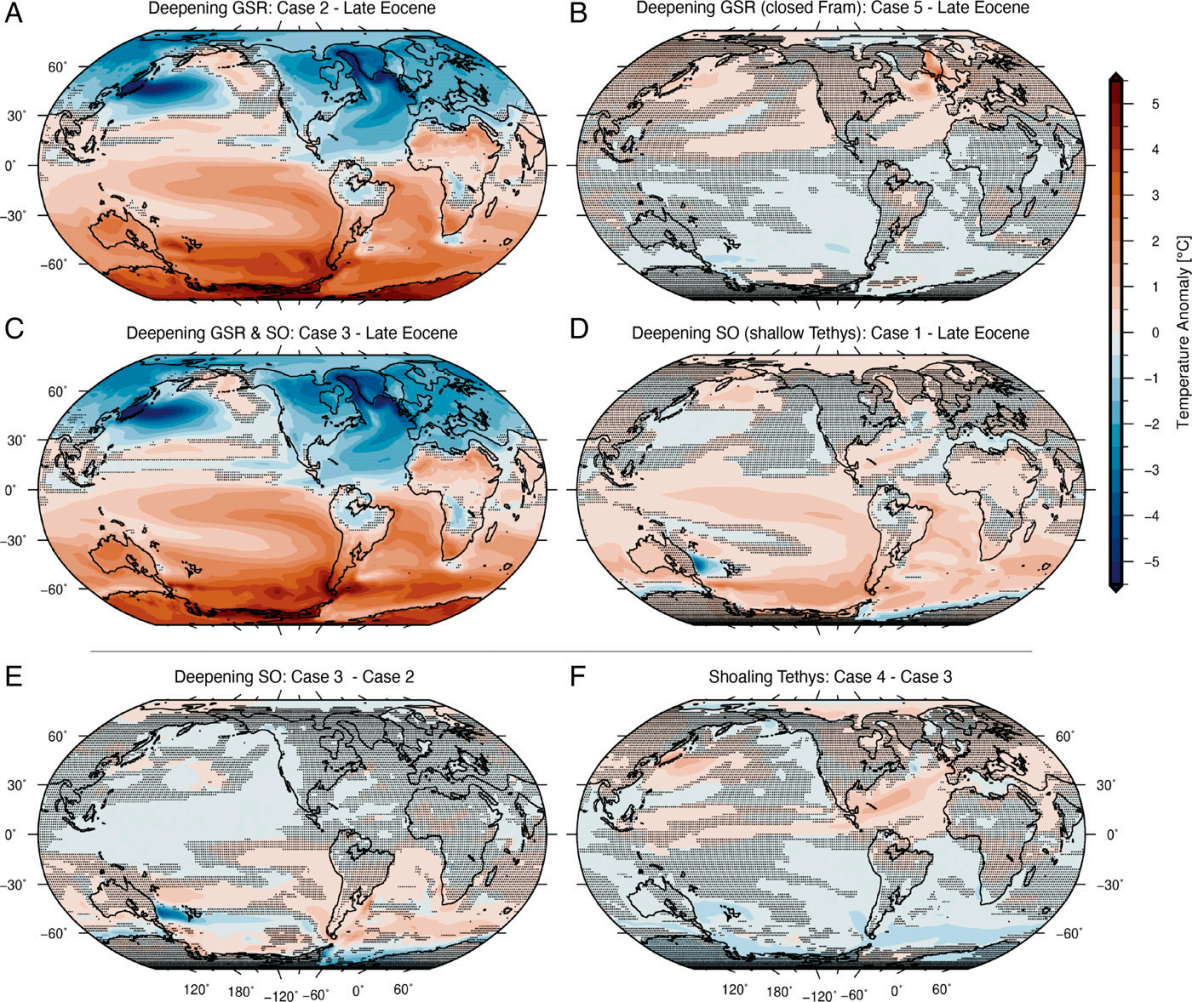
Surface air temperature anomalies in response to changes in oceanic gateways (100-y mean). Hashed regions mark areas where the temperature changes are statistically insignificant (*t* test). (*A*) Deepening the GSR with deep (∼140 m) Fram Strait (case 2, Late Eocene), (*B*) deepening the GSR with a very shallow (∼20 m) Fram Strait (case 5, Late Eocene), (*C*) deepening the GSR and the Southern Ocean gateways (case 3, Late Eocene), (*D*) deepening the Southern Ocean gateways and shoaling the Tethys Seaway (case 1, Late Eocene), (*E*) deepening the Southern Ocean Gateways (case 3 – case 2), and (*F*) shoaling the Tethys Seaway (case 4 – case 3).

With the Late Eocene paleogeographic configuration, the Arctic Ocean is much fresher than today (mean sea surface salinity is ∼20 PSU) due to the limited connection to the adjacent seas (Fig. 1*C*). This agrees with proposed periods of very fresh conditions in the Arctic Ocean in the Eocene (40). Fig. 3 shows how changes in the Atlantic–Arctic and Southern Ocean gateways (as indicated in Table 1) impact the climate under realistic Late Eocene (34 Ma) paleogeographic and climatic boundary conditions compared to the Late Eocene simulation.

**Fig. 4.**
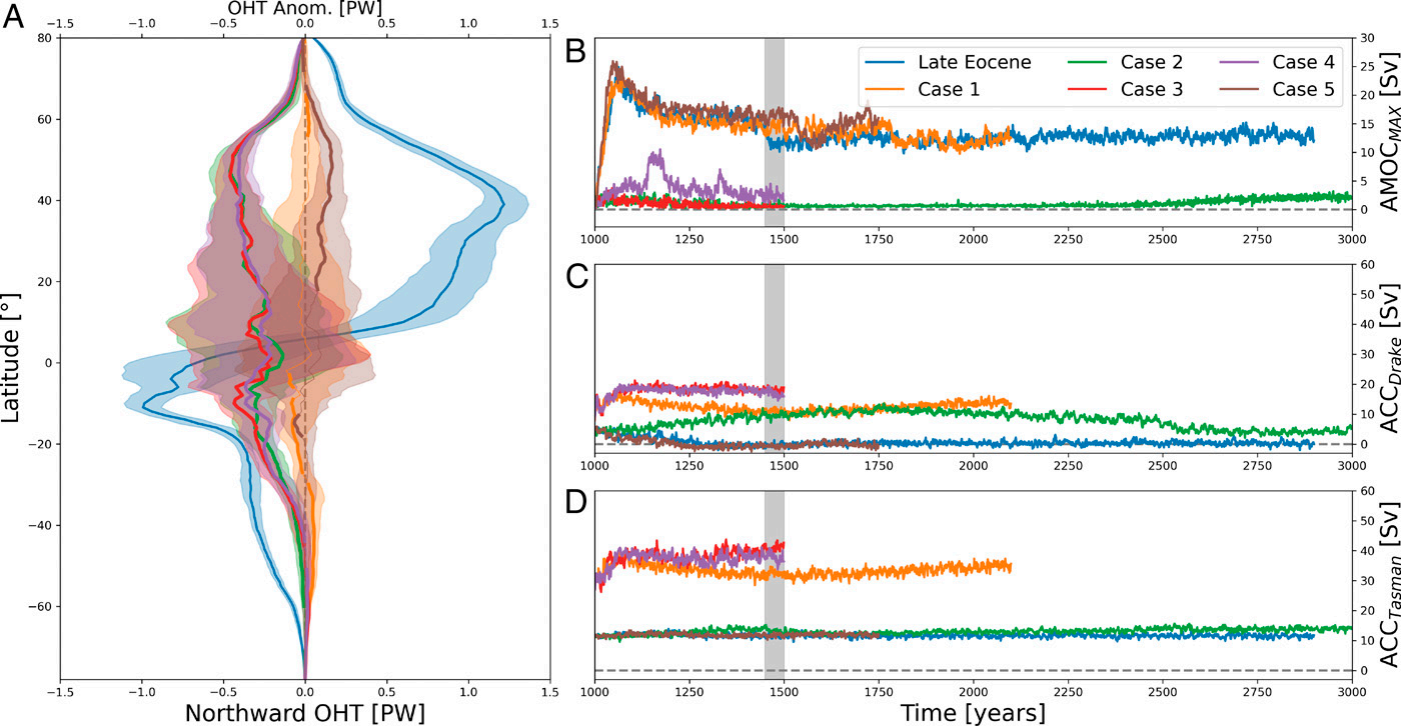
Ocean heat transport and circulation metrics. (*A*) Mean northward ocean heat transport for the Late Eocene simulation (blue, scale at the bottom) and the respective anomalies for other cases 1 to 5 (see Table 1; scale at the top) using years 1450 to 1500 (gray shading in *B*–*D*). (*B*) Evolution of the maximum strength of the AMOC. (*C*) Volume transport through the Drake Passage. (*D*) Volume transport through the Tasman Gateway.

**Fig. 5.**
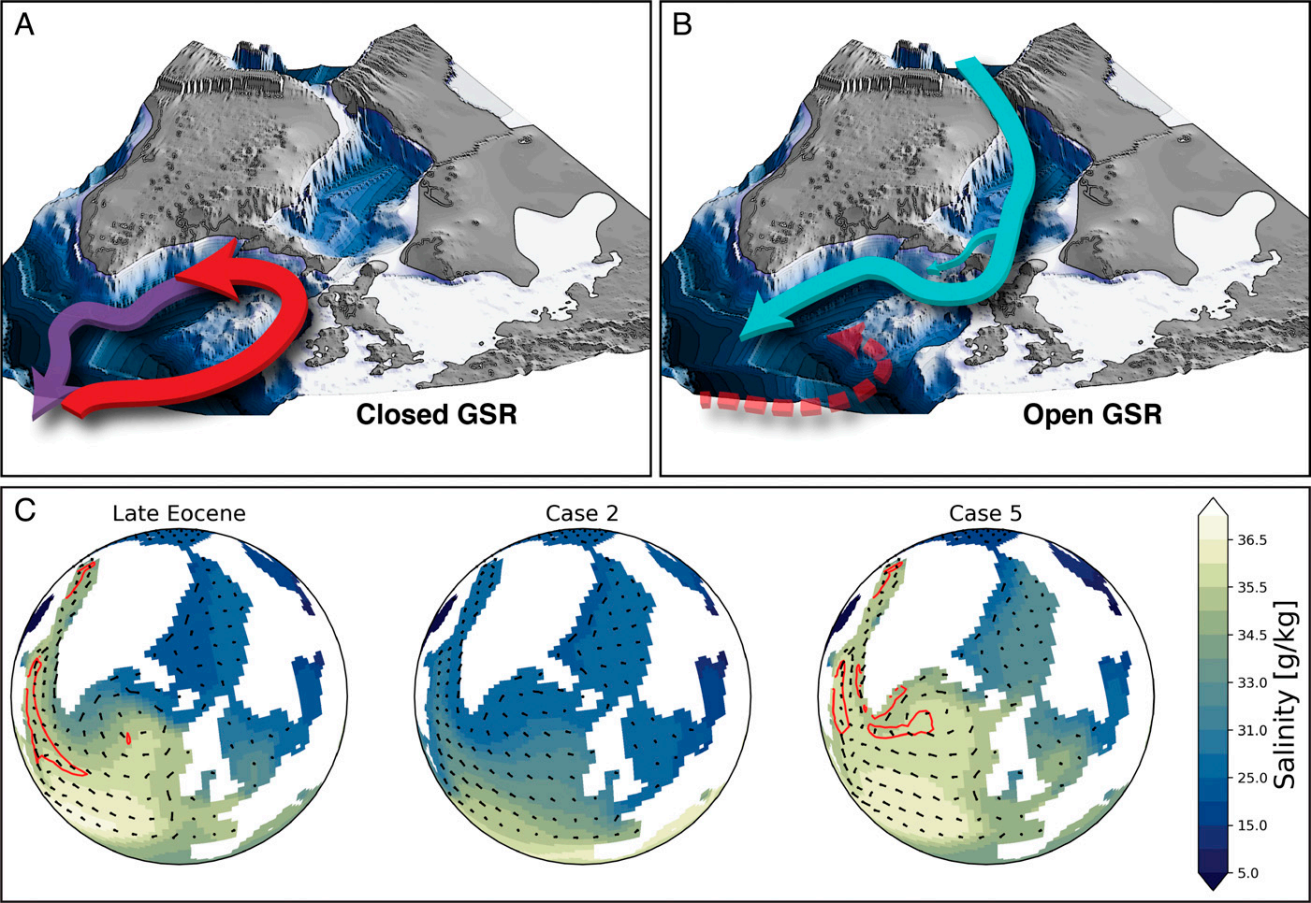
Changes in the North Atlantic Ocean paleobathymetry and ocean circulation. (*A*) Late Eocene with a shallow GSR. Red arrow indicates warm northward flow in the North Atlantic. Purple arrow indicates deep water formed south of the GSR (corresponds to the Late Eocene in *C*). (*B*) Deep GSR with a deep gateway through the FSC and a shallow strait across the Iceland Faroe Ridge. The proto–Fram Strait is a shallow passage to the Arctic. Cyan arrows indicate the southward flow of fresh Arctic waters preventing sinking in the North Atlantic. Dashed red arrow indicates a weak northward flow (corresponds to case 2 in *C*). (*C*) The 50-y (1450 to 1500) mean of the North Atlantic sea surface salinity for the Late Eocene simulation (closed GSR), case 2 (open GSR), and case 5 (open GSR and closed Fram Strait). Red lines mark the 500-m mixed layer contours. Black lines mark the flow direction.

Deepening the GSR (Fig. 3*A*) through the Faroe–Shetland Channel and the Iceland–Faroe Ridge allows for a fresh Arctic inflow to the North Atlantic that inhibits deep water formation there (Fig. 5). Without the deep water formation, both the overturning and the gyre circulations are weaker (Fig. 4 and *SI Appendix*, Fig. S5), transporting less heat northward (Fig. 4*A*), causing a significant cooling in the Northern Hemisphere that is centered over the North Atlantic (Fig. 3*A*). The climatic response to these changes in the North Atlantic ocean circulation is further intensified and communicated globally due to a well-understood atmospheric feedback (e.g., ref. 41 and references therein) of a southward shift of the atmospheric Hadley circulation (toward the warmer hemisphere) manifested as a southward shift of the tropical rain bands, i.e., the ITCZ (*SI Appendix*, Figs. S10 and S15). Note that with a deep GSR and cessation of the AMOC, the Antarctic continent warms considerably, even when the Southern Ocean gateways are open (see below).

The cessation of the AMOC in the experiments with a deep GSR (cases 2 to 4) requires a passage for fresh Arctic waters to the Nordic Seas (Fig. 4), either over the East Greenland Margin (cases 2 to 4) or through the Barents Sea (e.g., ref. 14). The Barents Sea was likely subaerial at this time (e.g., ref. 42), but our paleogeography model indicates that a shallow connection (∼140 m, marked as “deep” in Table 1 as this is the deepest of our tested configurations) may have existed along the East Greenland margin in the proto–Fram Strait (23). This connection between the Arctic Ocean and the Nordic Seas is supported by a recent mineralogical study on Ocean Drilling Program (ODP) Site 913 (43) with samples showing evidence of sea ice export from the Arctic Ocean to the Nordic Seas in the Mid-Eocene. Also, a recorded global mean sea level rise in the Late Eocene leading up the EOT (44) could have opened and maintained the Atlantic–Arctic connection.

An open GSR with a closed Fram Strait (Fig. 3*B*), however, extends the AMOC northward, warms the Nordic Seas, and leads to a slight increase of surface air temperatures over northern Eurasia but with little effect elsewhere. Therefore, the GSR depth fluctuations have little impact on the AMOC and global climate unless the proto–Fram Strait or the Barents Sea is partly open (as in Fig. 3*A*). This differs from the Early to Middle Eocene model of Vahlenkamp et al. (45) who simulated a decrease in Nordic Seas salinity and deep water formation, both when the proto–Fram Strait was closed (but GSR was shallow) and when the proto–Fram Strait was open (but GSR was deep, similar to our case 4). In our simulations, the GSR mainly limits the freshwater flow to the convective regions in the North Atlantic, rather than allowing for deep water formation in the Nordic Seas as in Vahlenkamp et al. (45).

Deepening the Southern Ocean gateways (Fig. 3 *C–E*) initiates a proto-ACC and a cooling of the sea surface along the coast of Antarctica. Although there is a transient 100-y-long drop in the surface air temperatures over the Antarctic continent (*SI Appendix*, Fig. S17), after 500 y of perturbation, temperatures over most of the Antarctic continent are not significantly different from the Late Eocene simulation. The proto-ACC is considerably weaker (∼10 to 15 Sv; Fig. 4*C*) than the present-day ACC at ∼150 Sv (46), although similar in strength to other recent model studies (47). A weak ACC could partly explain the lack of influence on Antarctic surface air temperatures and is expected in this configuration as the Southern Ocean gateways were narrower and shallower that today. However, a stronger ACC is not achieved even if we further deepen and widen the Southern Ocean gateways beyond realistic values in our model for that time (*SI Appendix*, Fig. S14). This suggests that opening the Southern Ocean gateways alone is not sufficient to initiate a strong ACC and that other mechanisms, such as the Tasman Gateway aligning with the westerlies (48) or the global Cenozoic cooling providing a stronger meridional temperature gradient (49), are needed for a strong ACC. Also, in contrast to studies using a modern tectonic configuration instead of one based on paleogeography (17) or an idealized topographic setup (50), we see no significant changes in the AMOC behavior and the Northern Hemisphere climate as a result of deepening the Southern Ocean gateways (case 1; Figs. 3*D* and 4*B*). Instead, the overturning in the North Atlantic is mostly separate from the overturning in the Southern Hemisphere. This is likely linked to the open Central American Seaway that allows both northern and southern sourced deep waters to spill to the Pacific instead of crossing the equator in the Atlantic Ocean.

Shoaling of the Tethys Seaway (Fig. 3*F*) leads to reduced freshwater transport from the Indo-Pacific to the Atlantic Ocean, a more saline North Atlantic, and a larger salinity difference between the Atlantic and the Pacific Ocean (case 4 − case 3; *SI Appendix*, Fig. S6*E*). A consequence of increasing salinity in the Atlantic Ocean could be an active AMOC even when the GSR is deep (as in case 4). There are periodic events of AMOC strengthening (case 4; Fig. 4*B* and *SI Appendix*, Fig. S3); however, the simulation does not stabilize with a strong active AMOC. The slight increase is enough to yield a weak but significant warming over the Northern Hemisphere. The combined effect of shoaling Tethys and deepening the SO gateways (Fig. 3*D*) is also a weak but significant warming over most of the global oceans, with little significant signal over land.

Finally, we tested the effect of decreasing atmospheric CO_2_ (Fig. 2 and *SI Appendix*, Figs. S2 and S3) by lowering the CO_2_ concentrations of the Late Eocene simulation and case 2 from 854.1 ppm (3 times preindustrial levels) to 569.4 ppm (2 times preindustrial levels). Lowering the CO_2_ cools the high latitudes (Fig. 2) in both cases. In the Late Eocene setup where the AMOC is active (shallow GSR), lowering CO_2_ enhances AMOC, whereas in case 2 where the AMOC is weak (deep GSR), lowering the CO_2_ also promotes deep water formation in the North Pacific Ocean, triggering an active PMOC (*SI Appendix*, Fig. S3).

## Discussion

The tectonic and geodynamic processes leading to the paleobathymetric changes in the oceanic gateways are generally regarded as important for climatic changes occurring on million-year timescales and longer. However, our results show that relatively small elevation changes (∼100 m), especially for the Atlantic–Arctic oceanic gateways, have large impacts on ocean circulation and climate. These changes can occur on significantly shorter timescales, even within the ∼400-kyr time interval of the EOT. In addition, several studies have shown that if oceanic gateways cross a certain threshold depth, there may be a distinct shift in the pattern of ocean circulation leading to geologically rapid climatic changes (e.g., refs. 45, 51, and 52). The plume-related bathymetric changes in the Northeast Atlantic are on the order of ∼200 m and could therefore have significant impacts on regional and global ocean circulation changes (14, 29, 45, 52). This is consistent with our simulations, which show the ocean and climate state before and after a realistic change in paleogeography. However, we cannot deduce from this study if the climatic changes occurred geologically instantly (millennial timescales), for example, from crossing some threshold, or gradually as a gateway opened or closed. The roles of key oceanic gateways in Eocene–Oligocene climatic changes are discussed below.

### The Role of the Atlantic–Arctic Gateways in the Eocene–Oligocene Cooling.

Variations in the Iceland mantle plume activity could have had a significant influence on the Eocene–Oligocene climate. Due to changing dynamic support from the Iceland mantle plume, the GSR shoaled in the Late Eocene before subsiding again in the Early Oligocene (22, 23). In general, high-latitude sites in both hemispheres show a mean cooling of ∼4.8 °C across the EOT (9). However, a recent estimate of Paleogene North Atlantic Ocean temperatures suggests that the temperature change across EOT was asymmetric and that the Northern Hemisphere cooled at least ∼400 kyr after the Southern Hemisphere cooling and the initial growth of Antarctic ice sheets (53). Our model results, backed up by proxy records and other modeling studies, show that the Late Eocene uplift and the Early Oligocene subsidence of the GSR could explain the global asymmetric cooling at the EOT, and we suggest the following scenario:

The GSR shoaling from the Middle to Late Eocene blocked the exchange of Arctic fresh water across the ridge. This strengthened the proto-AMOC and cooled the Southern Hemisphere (Fig. 4 *A* and *B*). Recent studies based on proxy data, such as δ^13^C (e.g., refs. 12 and 13), and modeling (e.g., refs. 14 and 17) support an onset of a proto-AMOC at this time (Late Eocene). A stronger AMOC in our simulations causes significant cooling of the Southern Hemisphere, due to a shift in meridional ocean heat transport, agreeing with the Southern Hemisphere SST proxies (9) and the start of Antarctic glaciations. A strengthening of the AMOC would also warm the Northern Hemisphere, which contradicts some of the temperature proxies (e.g., refs. 9 and 54). However, decreasing atmospheric CO_2_ could cancel the local warming caused by gateway-induced ocean circulation changes in the Northern Hemisphere. We see that reducing CO_2_ in our model experiments (Late Eocene–reduced CO_2_ and case 2–reduced CO_2_; *SI Appendix*, Figs. S2 and S3) cool the high northern latitudes with a similar magnitude to that induced by the Atlantic–Arctic gateways, which would explain why the AMOC-induced warming is not seen in the proxy record (see *Ocean Ventilation and CO_2_ Variations Facilitated by Gateway Changes*). Proxy evidence of the ITCZ moving northward during the EOT supports our interpretation of a strengthening AMOC (8).

Shortly after the EOT, as the Iceland plume weakened (22), the GSR subsided. Combined with a deepening of the Faroe Shetland Channel (FSC), this change in oceanic basin depth created a deep seaway in the Early Oligocene (23) (our deep gateway scenario, cases 2 to 5; Table 1), with a connection from the Arctic through the Nordic Seas to the North Atlantic. As a consequence, the AMOC collapsed, causing a significant cooling of the Northern Hemisphere. According to our simulations, when the AMOC is reduced (cases 2 to 4), the surface air temperatures over northern Eurasia cool significantly (generally by ∼1 to 5 °C; Fig. 3 *A* and *C*). The cooling is strongest just south of the GSR, were the surface temperatures cool by more than 5 °C. These results match the inferred Eocene–Oligocene temperature changes for the Northern Hemisphere (e.g., refs. 9, 10, and 54). SST proxies from ODP Sites 336 and 913 drilled north of the GSR (Fig. 1*B*) indicate a cooling of 2.2 °C and 6.7 °C, respectively (9). A decrease in bottom water temperatures (∼3 to 4 °C) for the Labrador Sea region has been reported for the 37.5 to 35 Myr time interval (ODP Site 647; ref. 13), and mean annual temperatures over Northern Europe decreased by ∼4 to 6 °C, according to data based on UK carbonate shells of freshwater gastropods (54). Terrestrially derived spore and pollen assemblages preserved in marine sediments in the Norwegian–Greenland Sea also indicate ∼5 °C cooling from the Late Eocene to Early Oligocene, although this cooling is confined to the winter months (10). Note that the simulated SST cooling (∼1.7 °C at site 336 and ∼2.3 °C at site 913; Fig. 1*B*) resulting from deepening the GSR is less than the Liu et al. (9) proxy estimates. We suggest that the additional recorded EOT cooling (7, 55) could have been caused by decreasing CO_2_, where the cooling at both sites is ∼6 °C when combining a 33% reduction in CO_2_ with deepening the GSR (case 2–reduced CO_2_). With the open Atlantic–Arctic connection, the model shows a warming of the Southern Hemisphere (Fig. 3 *A* and *C*), which could partly explain the rapid recovery to a warmer climate at the end of the EOT and the termination of Antarctic ice growth (e.g., ref. 1).

### Ocean Ventilation and CO_2_ Variations Facilitated by Gateway Changes.

Our model results show distinct surface temperature changes over Antarctica along with the Southern Hemispheric cooling caused by shoaling the Atlantic–Arctic oceanic gateways (Figs. 3 and 4). However, these bathymetry-driven changes in ocean circulation alone may not produce a large enough cooling to trigger large-scale ice sheet growth. We also observe a distinct cooling when reducing CO_2_ in our simulations, and the combined effect of gateway changes and decreasing CO_2_ gives the strongest climatic changes in our simulations, especially in the high latitudes. Therefore, to explain the strong cooling observed in the proxy record, the changes in the ocean circulation were likely accompanied by reduced atmospheric pCO_2_.

We observe a significant increase in winter snow accumulation in the Northern Hemisphere when deepening the GSR (*SI Appendix*, Fig. S11*B*). This trend is further intensified by reducing the CO_2_ (*SI Appendix*, Fig. S12) and points toward more favorable conditions for ice sheet growth, supported by Eocene–Oligocene ice rafted debris in Norwegian–Greenland Sea sediments, likely originating from East Greenland continental ice sheets at this time (11). Although continental ice growth cannot directly be inferred from our simulations, and little snow from the winter survives the summer (*SI Appendix*, Fig. S13), the trends we observe indicate that Northern Hemisphere ice sheets are more likely to accumulate with the right paleogeographic boundary conditions, under higher pCO_2_ concentrations than previously modeled (55). The Antarctic snow depths decrease at lower altitudes due to the Southern Hemisphere warming caused by deepening the GSR, but we see an increase inland on higher altitudes caused by increased precipitation (*SI Appendix*, Figs. S10*B* and S11*B*). Reducing CO_2_ accumulates more snow at lower altitudes and along the coastline but reduces the higher-altitude snow inland (*SI Appendix*, Fig. S12).

One mechanism explaining the drawdown of pCO_2_ at that time is the stronger ocean ventilation (as found in our simulations) combined with enhanced biological productivity. The scenario we suggest is a switch to ventilation in the North Atlantic (due to the uplift of the GSR) reduced the total ocean carbon sink before the EOT, while cooling down the Southern Hemisphere. In the Early Oligocene the GSR deepened, and ventilation is again dominated by the Southern Ocean and the North Pacific.

**Fig. 6.**
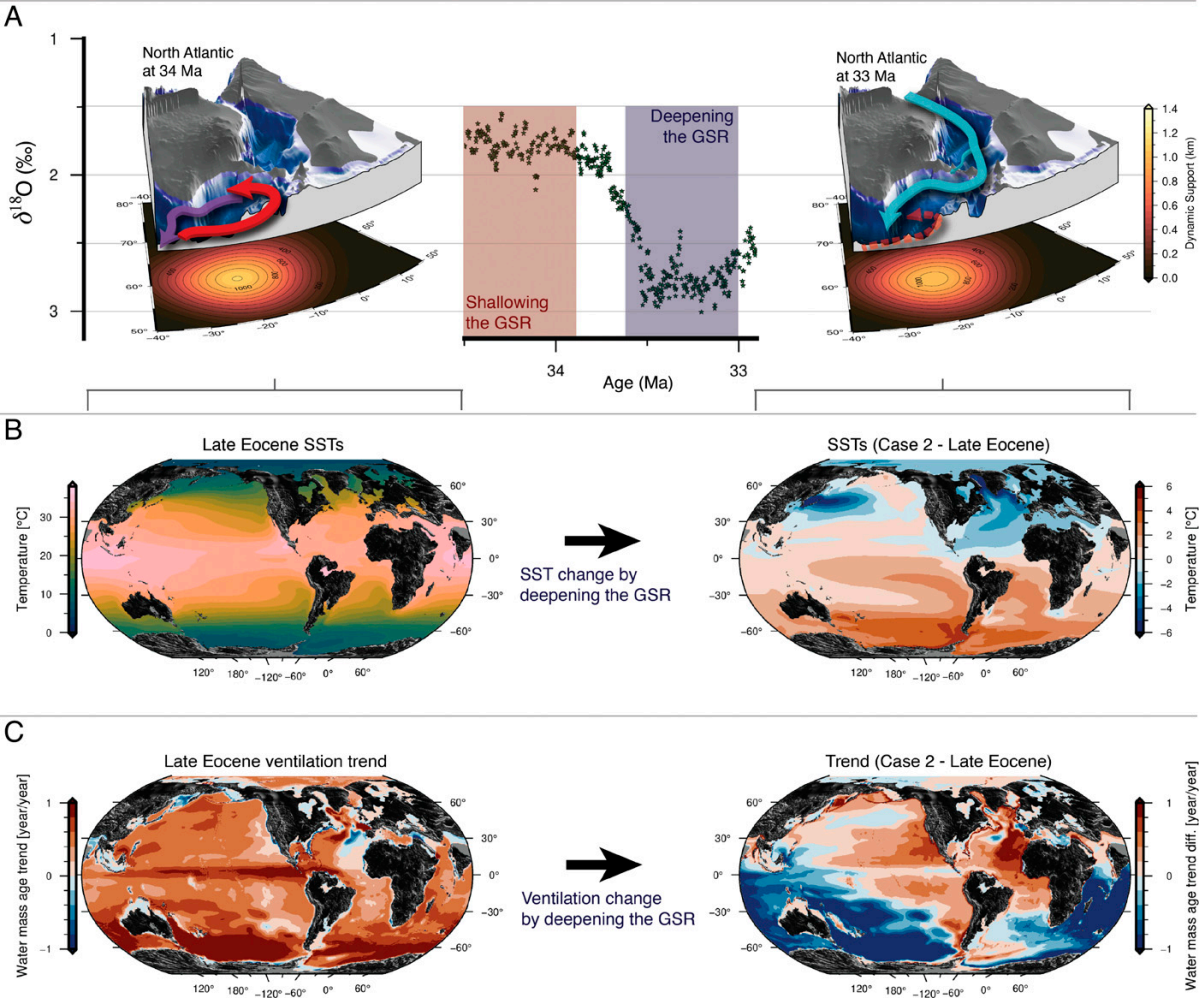
Changes in Iceland mantle plume activity leading to changes in SSTs and ocean ventilation. (*A*) The benthic oxygen isotope values ($\delta^{18}O$) across the EOT (6) and the paleogeographic configuration for 34 Ma (*Left*) and 33 Ma (*Right*), with the dynamic support from the Iceland plume (23) and sketched ocean circulation for the respective gateway configurations based on this study. (*B*) The change in SSTs forms as a result of deepening the GSR (a likely paleogeographic change from 34 to 33 Ma). (*C*) Change in ocean ventilation is measured as the trend in the vertical mean water mass age when deepening the GSR.

When the GSR is deep, our simulations show a shift in the ocean ventilation away from the North Atlantic to the Southern Ocean and North Pacific, thereby increasing global ocean ventilation by 20 to 34% (Fig. 6). This would have transported deep, nutrient-rich waters to the surface, enhancing biological productivity and CO_2_ drawdown, and could thereby have contributed to the decrease in the atmospheric pCO_2_ from ∼1,000 ppm during the Late Eocene toward the EOT, stabilizing at ∼750 ppm in the Early Oligocene (7, 56). The CO_2_ drawdown could have been further enhanced by increased continental weathering as we simulate a small (1%), but statistically significant, increase in global land precipitation as the ITCZ moves southward when the AMOC collapses (*SI Appendix*, Fig. S10).

The atmospheric pCO_2_ contribution from ocean ventilation across the EOT is also supported by local estimates of atmospheric CO_2_ levels from boron isotopes across the EOT (Tz in Fig. 1) (57), which suggest a decrease in CO_2_ values before and during the first EOT phase, followed by a sharp recovery to pretransition values from ∼33.6 Ma to 33.4 Ma and a more gradual decline into the Oligocene (57).

One caveat in our simulations is the deep tidal mixing which could have been enhanced during the Eocene (58) but is not taken into account in our model. Stronger mixing would have enhanced the overturning in the Southern Ocean and in the North Pacific, but freshwater flux from the Arctic to the North Atlantic causes a large enough salinity change (>10 g/kg) so that changes in the Atlantic–Arctic gateways would have still dominated the deep-water formation there.

## Summary

Our simulations can explain the observed hemispheric asymmetries in climate changes across the EOT as the temporal GSR uplift in the Late Eocene strengthened the AMOC and cooled the Southern Hemisphere, thereby contributing to the initial glaciation of the Antarctic continent. Later deepening of the GSR, in combination with a shallow seaway to the Arctic Ocean, caused a cessation of the AMOC and cooling of the Northern Hemisphere. We show that relatively small depth variations of the Atlantic–Arctic oceanic gateways may have large implications for the dynamics of regional and global ocean circulation.

Overall, the bathymetric changes of the GSR and the presence of a shallow proto–Fram Strait have the most significant impacts on the Eocene–Oligocene climate compared to bathymetric changes to the Southern Ocean Gateways and the Tethys Seaway.

Based on our simulations and published proxy records, we suggest that the AMOC strengthened in the Late Eocene due to increased dynamic support from the Iceland Plume. This allowed for an active AMOC in the North Atlantic, with a significant spilling of the deep waters to the Pacific through the Central American Seaway. This moved the ITCZ northward, as observed in the proxy records, and significantly cooled the Southern Hemisphere, facilitating the initial glaciations of Antarctica.

At the end of the EOT, deepening Atlantic–Arctic gateways enables fresh Arctic waters to flow across the GSR preventing deep water formation south of the ridge, ultimately leading to a collapse of the AMOC (Fig. 4). This causes significant cooling of the Northern Hemisphere, shifting the ITCZ south and allowing for CO_2_ drawdown by enhanced ventilation in the Southern Ocean and North Pacific. The ventilation could have been further enhanced by Southern Ocean gateway openings, although our results show that they are not necessary for this scenario.

The decrease in CO_2_ causes hemispheric symmetric cooling in our simulations, in contrast to the hemispheric asymmetric cooling caused by changes to the Atlantic–Arctic oceanic gateways. Therefore, decreasing CO_2_ while deepening (shallowing) the Atlantic–Arctic oceanic gateways enhances the simulated cooling in the Northern (Southern) Hemisphere and reduces warming in the Southern (Northern) Hemisphere.

Opening the Southern Ocean gateways enables the flow of a proto-ACC, which is considerably weaker than the present (Fig. 4). This causes a slight cooling along the coast of Antarctica, but it is not sufficient to compensate for the warming caused by deepening the Atlantic–Arctic oceanic gateways. Shallowing the Tethys Seaway controls the salinity difference between the Atlantic Ocean (relatively saline) and Pacific Ocean (relatively fresh), and its shoaling causes a weak increase of the AMOC.

## Materials and Methods

To estimate the climatic impact of the bathymetric changes during EOT, we implement the paleogeographic reconstruction of Straume et al. (23) in the NorESM. In this study we use the NorESM-F version of the model (38), which has been previously used for both paleosimulations and historical and future projections in the Coupled Model Intercomparison Project (CMIP) context (38, 59–61). NorESM-F can be considered a Community Earth System Model (CESM) variant and consists of the Community Atmosphere Model, Community Land Model, Community Sea Ice Model, and Bergen Layered Ocean Model (formerly the Miami Isopycnic Coordinate Ocean Model [MICOM]). The atmosphere and land models are configured on 2° resolution, whereas the ocean and sea ice share a tripolar grid configured on a nominal 1° resolution. The ocean grid is refined to be 1/4° in latitude over the tropics to capture the narrow tropical current systems; in addition, the two northern singularities are placed around 40°N in North America and eastern Asia, enhancing the resolution around the Gulf Stream and Kuroshio regions. The individual components are coupled together using Common Infrastructure for Modeling the Earth (CIME). The ocean biogeochemistry is not active in these simulations.

The model is configured on 51 potential density layers (referenced to 2,000 dbar) with two-layer bulk mixed layer at the top (in total 53 vertical levels). The layered model is well equipped to simulate flow over (even steep) bathymetry that is of interest here. When making a deep perturbation to the bathymetry we stretch the last existing layer to the bottom, and when we make a shallow perturbation to the bathymetry, we set the thickness of the layers below the new bottom depth to zero and adjust the bottom thickness of the bottommost layer so that the overall water column thickness matches the bottom depth. This procedure is nonconserving, but because the change is introduced in a small number of grid cells, it does not affect the overall solution. The changes made here also do not change the land–sea mask making the technical implementation easy.

The model is configured similar to the preindustrial control simulations in CMIP5, except the atmospheric CO_2_, which is taken to be 854.1 ppm (3 times the preindustrial control level) The atmosphere and the ocean are configured using the paleotopography of Straume et al. (23) and initialized from rest with the ocean initial conditions taken to be an idealized zonal mean that is 10° warmer than the present-day ocean. This decision was motivated by the results of Hutchinson et al. (14), who show that their deep ocean temperatures are around 10 °C at the end of their simulations.

The model is spun up for 1,000 y, after which the simulation reaches a semiequilibrium (*SI Appendix*, Fig. S2). At that point we initialize all the perturbation experiments and run them for another 500 y. Ideally, the simulations would have been run for several thousands of years, but the current setup is too expensive for that given the multiple perturbations. We tested some of the perturbations already after 500 y of spin-up, and the results were very similar to those presented here so we do not believe a longer spin-up would lead to qualitatively different results.

We compare the model SSTs from the Late Eocene simulation to the proxy compilation of Hutchinson et al. (37). We used the present-day proxy locations and the paleomagnetic reference frame of Torsvik et al. (62) to get the paleolocations of the drill sites consistent with our paleogeographic reconstructions.

## Supplementary Material

Supplementary File

## Data Availability

The NorESM output will be available at the NIRD Research Data Archive from spring 2022. The output is currently available in Zenodo at https://doi.org/10.5281/zenodo.6399300.
