# Supplementary material for: Climate transition at the Eocene–Oligocene influenced by bathymetric changes to the Atlantic–Arctic oceanic gateways
Source: Proc Natl Acad Sci U S A. 2022 Apr 21;119(17):e2115346119. doi: 10.1073/pnas.2115346119 (PMC9169914; doi:10.1073/pnas.2115346119)
Supplement: Supplementary File [file pnas.2115346119.sapp.pdf]

PNAS
www.pnas.org

Supplementary Material for

Climate transition at the Eocene – Oligocene influenced by bathymetric changes to the Atlantic – Arctic oceanic gateways

E. O. Straume[1,4], A. Nummelin[1,3], C. Gaina[1,5], K. H. Nisancioglu[1,2]

[1] Centre for Earth Evolution and Dynamics, Department of Geosciences, University of Oslo, Norway.

[2] Department of Earth Scineces, University of Bergen, Norway.

[3] NORCE Norwegian Research Centre AS, Bjerknes Centre for Climate Research

[4] The Jackson School of Geosciences, University of Texas at Austin, USA

[5] School of Earth and Atmospheric Sciences, Queensland University of Technology, Australia

* Eivind O. Straume
Email: e.o.straume@geo.uio.no

This PDF file includes:

Supplementary text
Section 1: NE Atlantic paleogeography
Section 2: Model configuration and spinup
Section 3: Description of the sensitivity experiments
Section 4: Additional experiments: short runs

Supplementary figures
Figure S1: NE Atlantic paleogeography
Figure S2: Spin-up time series
Figure S3: Spin-up time series ocean circulation
Figure S4: Zonal mean temperatures
Figure S5: Ocean circulation and flow trajectories
Figure S6: Sea Surface Salinity (SSS) anomalies
Figure S7: Sea Surface Temperature (SST) anomalies
Figure S8: Ventilation trend in the vertical mean water mass age
Figure S9: Surface Temperature Anomalies
Figure S10: Mean annual precipitation anomalies
Figure S11: Snow depth anomalies in winter (gateways)
Figure S12: Snow depth anomalies in winter (gateways and reduced CO_2)
Figure S13: Snow depth anomalies in summer (gateways and reduced CO_2)
Figure S14: Circulation metrics and testing of additional deepening of the Southern Ocean gateways
Figure S15: Zonal mean precipitation.
Figure S16: Vertical section of change in global zonal mean salinity
Figure S17: Antarctic land surface temperature for different seasons

S1 NE Atlantic paleogeography

We use the paleogeography model of Straume et al (2020) for 34 Ma. We modify this model for the Tethys Seaway, Southern Ocean gateways, and the Atlantic – Arctic oceanic gateways, so that our deep and shallow gateway scenarios represent the max/min elevation possible at 34 Ma. For the Atlantic – Arctic oceanic gateways the preferred model is shown in Figure S1. The shallow scenario implemented in the model for the Greenland-Scotland Ridge is similar to the reconstruction shown in Figure 2, but with a shallower Faroe – Shetland Channel. The deep scenario has a shallow seaway over the Iceland – Faroe Ridge, and deeper Faroe – Shetland Channel, which is similar to the 26 Ma reconstruction in Figure S1. For the Fram Strait the deep scenario is exactly as shown for 34 Ma (Fig. S1), while in the shallow scenario the gateway is closed like in the 38 Ma reconstruction.

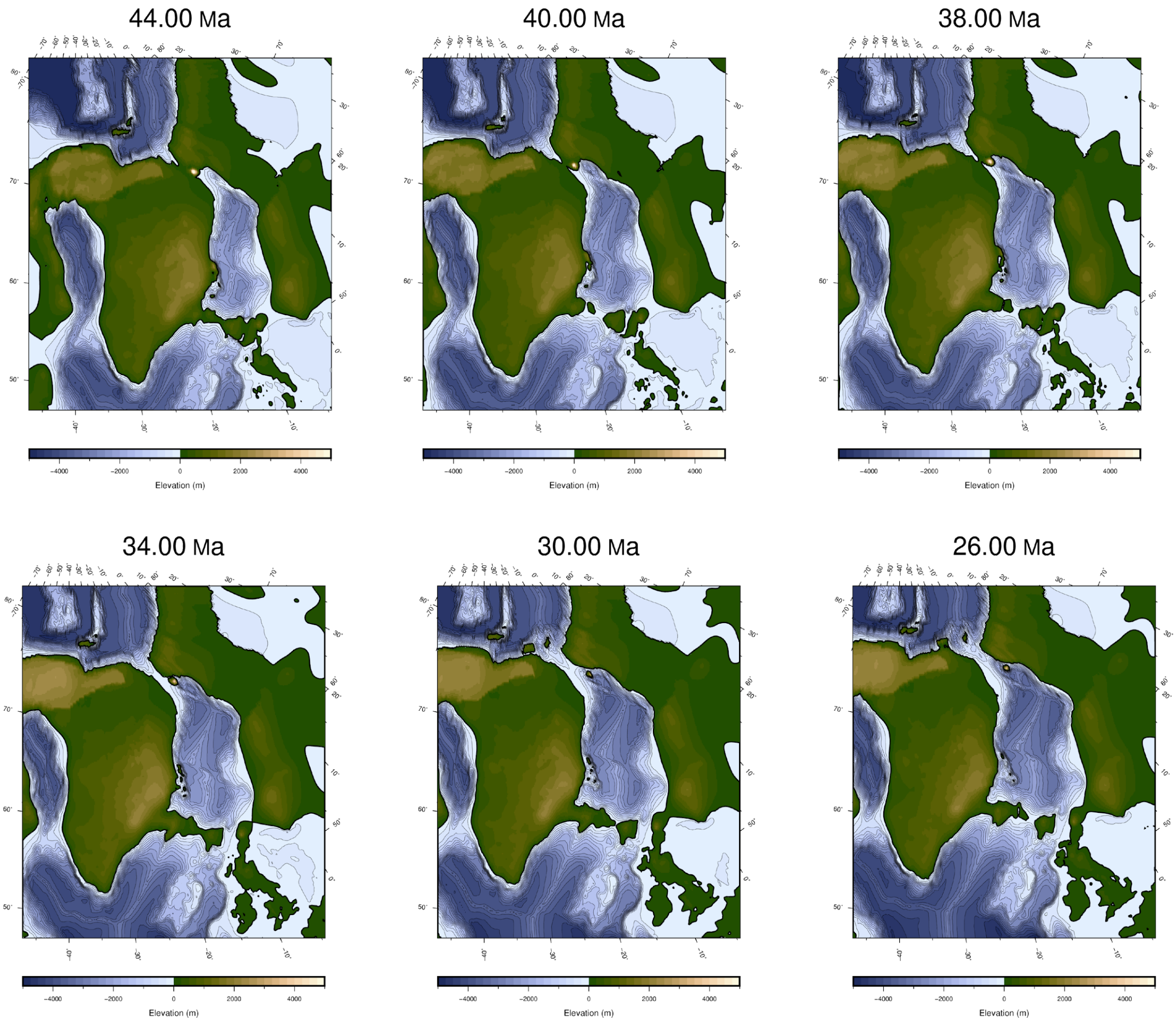

Figure S1: Paleogeography of the NE Atlantic region from 44 – 26 Ma (Straume et al. 2020).

S2 Model configuration and spinup

We use the NorESM-F (Guo et al. 2019) that is configured on a tripolar grid with an ocean domain that has a nominal 1° horizontal grid size that is refined in the tropics to be 0.25° in latitude in order to better capture the alternating zonal jets near the equator. The ocean model uses 51 potential density layers that are connected to two level bulk mixed layer. We use the same reference densities as used for the CMIP5/6 simulations. The model starts from rest using an idealized zonal mean profile which is constructed as follows: we begin from the World Ocean Atlas temperature and salinity climatology for 1981-2010, we first add 10°C to the temperature field everywhere and then calculate the average temperature and salinity profiles in the polar (poleward of 60°) and equatorial regions (equatorward of 30°). We then linearly interpolate meridionally to yield a complete zonal mean temperature and salinity profiles. The addition of 10°C to the temperature field globally is ad-hoc, but it is motivated by other

studies who have found that the global mean temperature at the EO boundary was close to 10°C (Hutchinson et al., 2019).

The spinup corresponds to the bathymetric setup of Case 2. We run the simulation for 1000 years at which point the simulation has reached a semi-equilibrium in terms of circulation metrics although small top-of-the-atmosphere radiative imbalance persist which causes small positive temperature drift (Figure S2-S3). For each perturbation we keep the land-sea mask and only modify the ocean modes restarts file as follows: for each case we create a dummy case with modified bathymetry and run it for 1 month starting from the initial conditions. We then replace the information in the original restart file (end of spinup) at the locations where the bathymetry was modified. This is an ad-hoc approach which does not conserve energy (kinetic, potential or heat). However, we do not expect that this ad-hoc approach affects the results as we apply the changes only in few grid cells and run the simulations for 500 years. The perturbations are also large compared to the drift that is left in the spinup. We also tested the perturbations starting from 500 years of the spinup (not shown) which lead to qualitatively similar response to the ones documented here. Therefore, we think that qualitatively the responses documented here are robust, but the quantitative results are bound to be model dependent.

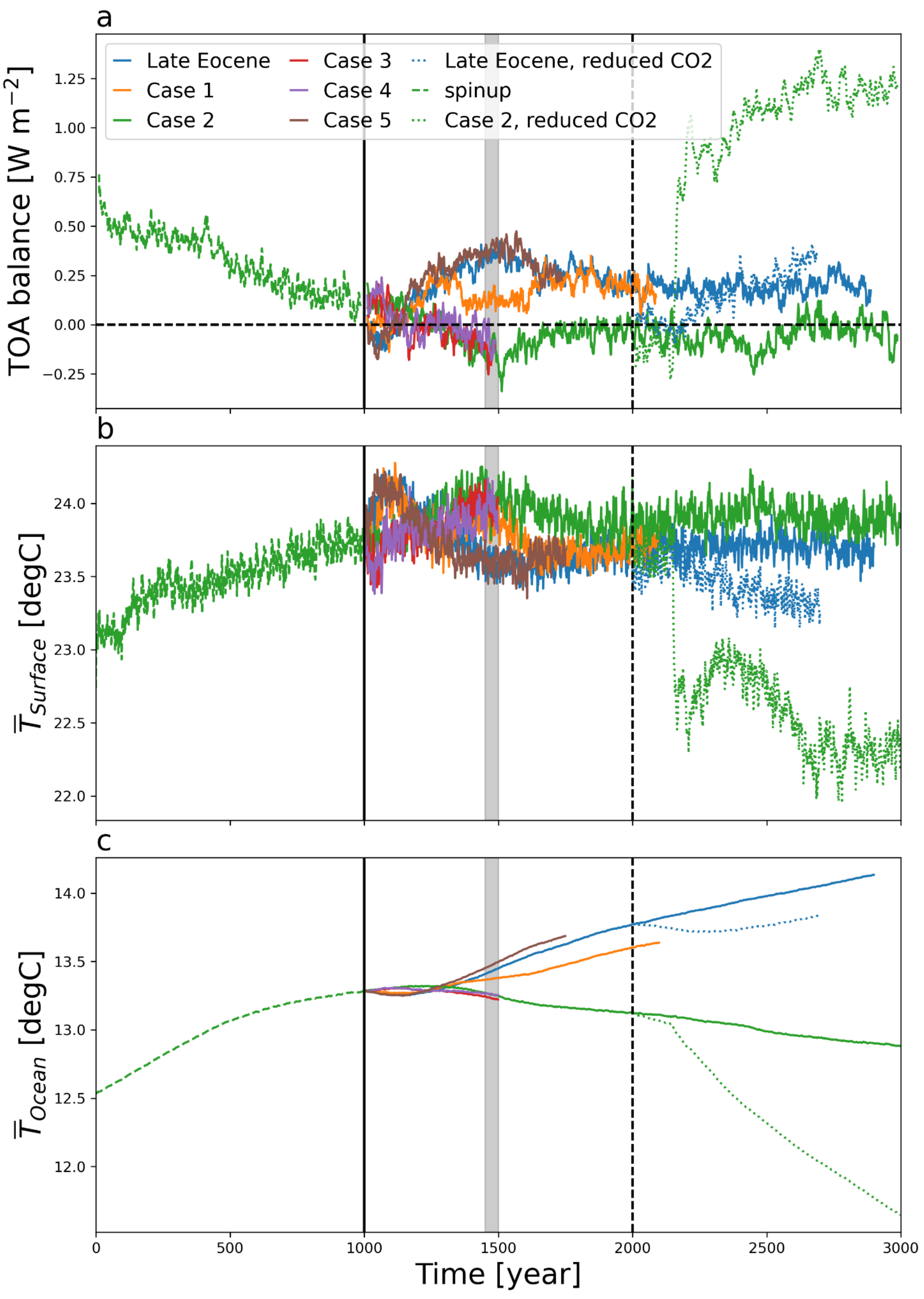

Figure S2. Time series of (a) top-of-the-atmosphere (TOA) radiative imbalance (positive up), (b) global mean surface air temperature (SAT), and (c) mass mean ocean temperature (T). Note that the same colors indicate identical paleogeographic setup i.e., the model spin up is done using the setup dubbed ‘Case 2’. Vertical solid line denotes the end of the spinup, vertical gray shading the period used for most of the analysis, and vertical dashed line the time at which the reduced CO2 cases are initiated.

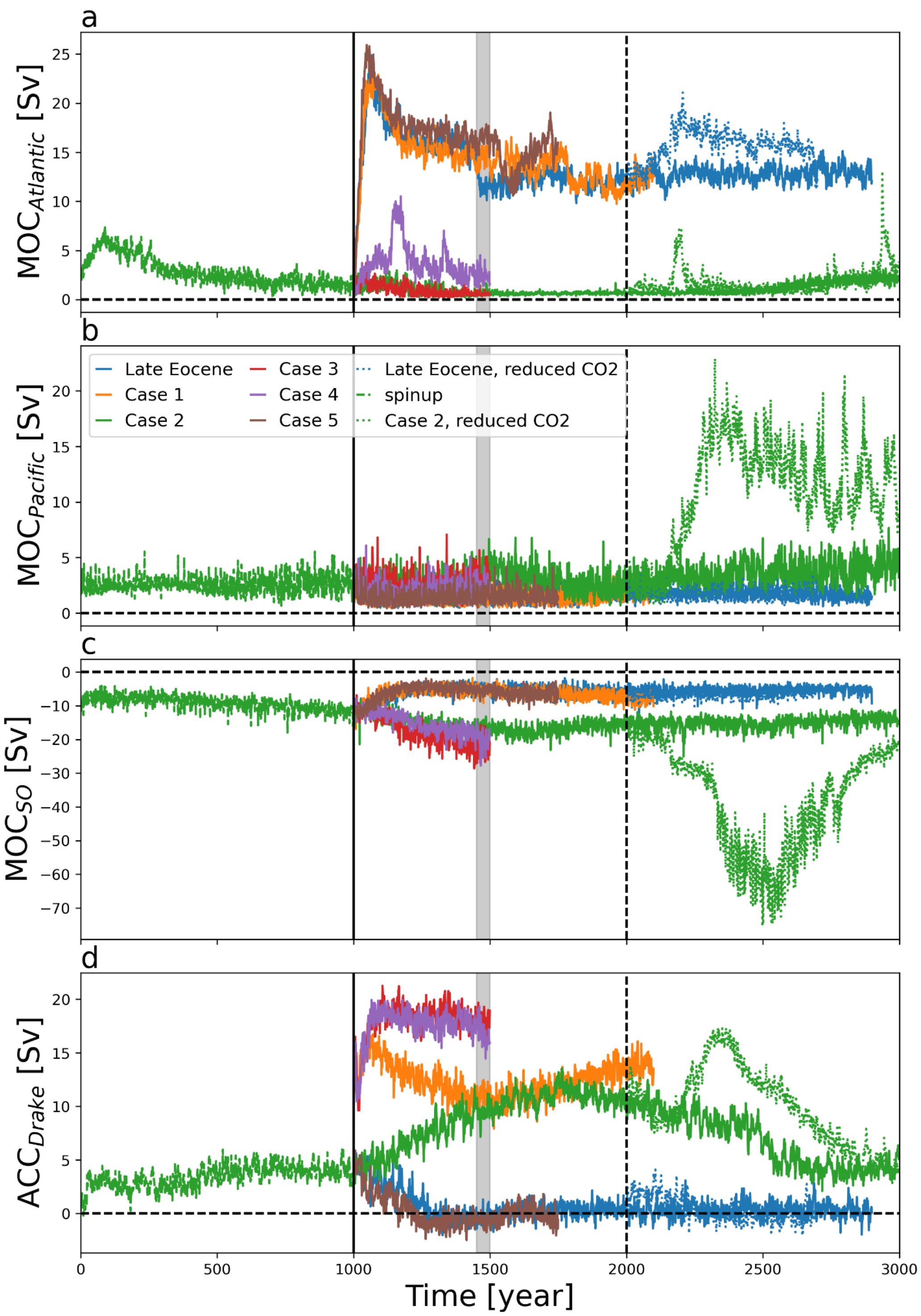

Figure S3. Time series of maximum Meridional Overturning Circulation (MOC) in (a) the North Atlantic, (b) the North Pacific, and (c) the Southern Ocean, as well as (d) a timeseries of volume transport through the Drake passage - a measure for the Antarctic Circumpolar Current (ACC) strength. Note that the same colors indicate identical paleogeographic setup i.e. the model spin up is done using the setup dubbed 'Case 2'. Vertical solid line denotes the end of the spinup, vertical gray shading the period used for most of the analysis, and vertical dashed line the time at which the reduced CO2 cases are initiated.

S3 The Sensitivity experiments

Late Eocene control: *Shallow GSR, Shallow Southern, Deep Tethys seaway.*

The Late Eocene simulation has an active AMOC (Fig. S3) which sustains a warm Northern Hemisphere. The Southern Ocean gateways are shallow, and there is no ACC in this configuration, which is sustaining a warm Antarctic continent. The Tethys Seaway is open and deep, contributing to the small salinity differences between the Atlantic and the Pacific Ocean (Fig. S6). For this paleogeographic setup, we have also done an additional simulation with reduced atmospheric CO2 concentration (2 times the pre-industrial instead of the original 3 times the pre-industrial).

Case 1 *Shallow GSR, Deep Southern Ocean, Shallow Tethys seaway*

Opening the Southern Ocean gateways initiates a proto-ACC cooling the SSTs around Antarctica (Fig. S7). This proto-ACC is much weaker than the present (Fig. S3) and is not enough to cause significant cooling of the surface temperatures on the Antarctic continent (Fig. S9). Opening the Southern Ocean gateways has little effect on the AMOC and the Northern Hemisphere climate.

Case 2 *Deep GSR, Shallow Southern Ocean, Deep Tethys seaway*

Deepening the GSR enables fresh Arctic waters to flow south across the ridge resulting in a freshening of the North Atlantic Ocean (Fig. S6). There is an associated salinity increase in the Arctic Ocean, North Pacific and South Atlantic oceans. The freshening of the North Atlantic shuts down the AMOC which causes significant cooling of the northern hemisphere SSTs (Fig. S7) and surface temperatures (Fig. S9). The Southern Hemisphere SSTs warms and the ITCZ shifts southward (Fig. S10). The surface temperatures in the southern hemisphere warm accordingly and there is warming of the Antarctic continent (Fig. S9). For this paleogeographic setup, we have also done an additional simulation with reduced atmospheric CO2 concentration (2 times the pre-industrial instead of the original 3 times the pre-industrial).

Case 3 *Deep GSR, Deep Southern Ocean, Deep Tethys seaway*

Deepening the Southern Ocean and the GSR cause cooling of the Northern Hemisphere (Fig. S9). The Southern Hemisphere warms as the warming caused by deepening the GSR is stronger than the weak cooling caused by deepening the Drake Passage and Tasman Gateway (Fig. S9).

Case 4 *Deep GSR, Deep Southern Ocean, Shallow Tethys seaway*

Shallowing the Tethys Seaway increases the salinity in the North Atlantic compared to case 3 (Fig. S6), but there are no significant surface temperature responses over land (Fig. S9).

Case 5 *Deep GSR, Shallow Southern Ocean, Deep Tethys seaway,* ***closed Fram Strait***

The gateway configuration is similar to the Late Eocene case, only here the Fram Strait is closed instead of the GSR. Surface climate is similar in the two cases, although deepening Fram Strait instead of GSR leads to slightly colder North Atlantic (Fig. S9d). Therefore, the results suggest that Fram Strait is more effective than GSR in controlling the North Atlantic Salinity and AMOC state, but the differences are small and concentrated in the Northern North Atlantic

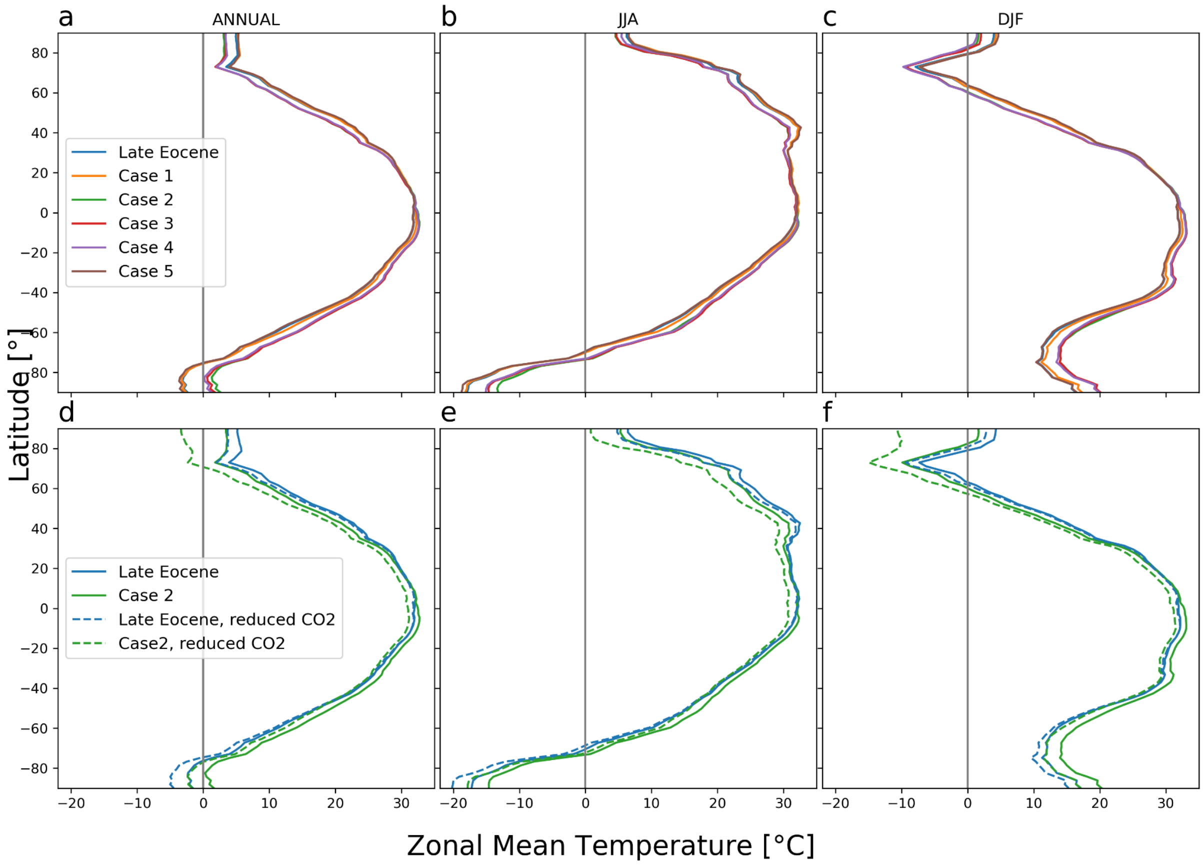

Figure S4. Zonal mean temperature averaged over years 1450-1500 for all cases and averaged over 2650-2700 for Late Eocene and Case 2 as well as their counterparts with reduced CO_2 concentrations: annual mean (a,d), June-August mean (b,e), December-February mean (c,f).

S4 Additional experiments: short simulations

Two of the perturbation cases were terminated after 200 years. These cases were terminated either as they reached a stable state or as they showed similar oceanographic and climatic sensitivity as some of the cases ran for 500 years and were therefore terminated to save computational resources.

Short simulation 1: (*Deep GSR, Deep Southern, Deep Tethys, Closed Fram Strait)*

The preliminary results were similar to case 5 in the sense that there are no considerable changes to the AMOC when deepening the GSR and the Fram Strait is closed. Also, effects of changing the Southern Ocean and Tethys Seaway were the same as for the cases with closed GSR.

Short simulation 2: (*Shallow GSR, Shallow Southern, Shallow Tethys)*

After 200 years this showed a similar response as the late Eocene control run. However, there were slightly higher salinities in the Atlantic Ocean, but this did not have significant impacts on the AMOC.

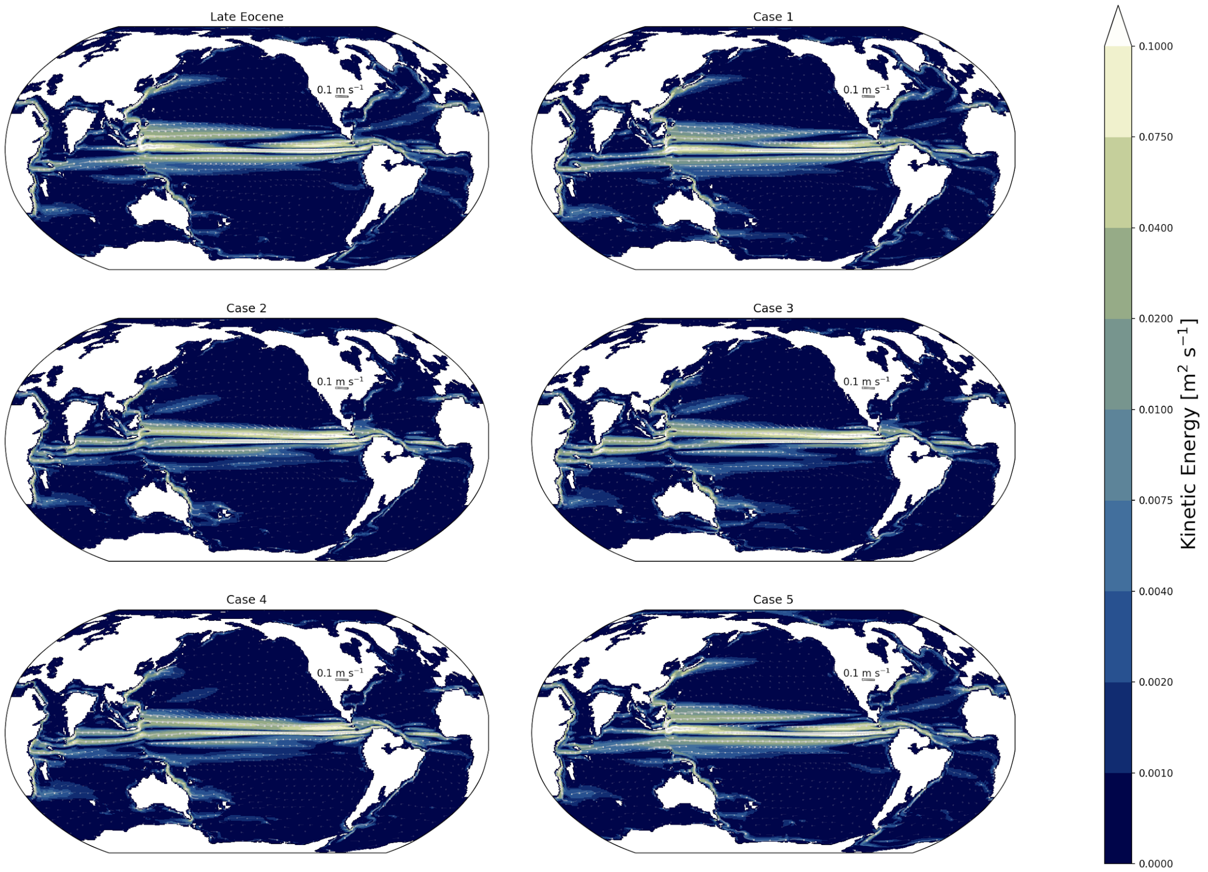

Figure S5: Ocean circulation and flow trajectories at 50 m depth averaged over years 1450-1500. The regions of high kinetic energy highlight the general ocean circulation pattern for the different cases. See results section in the main manuscript for description of the circulation pattern.

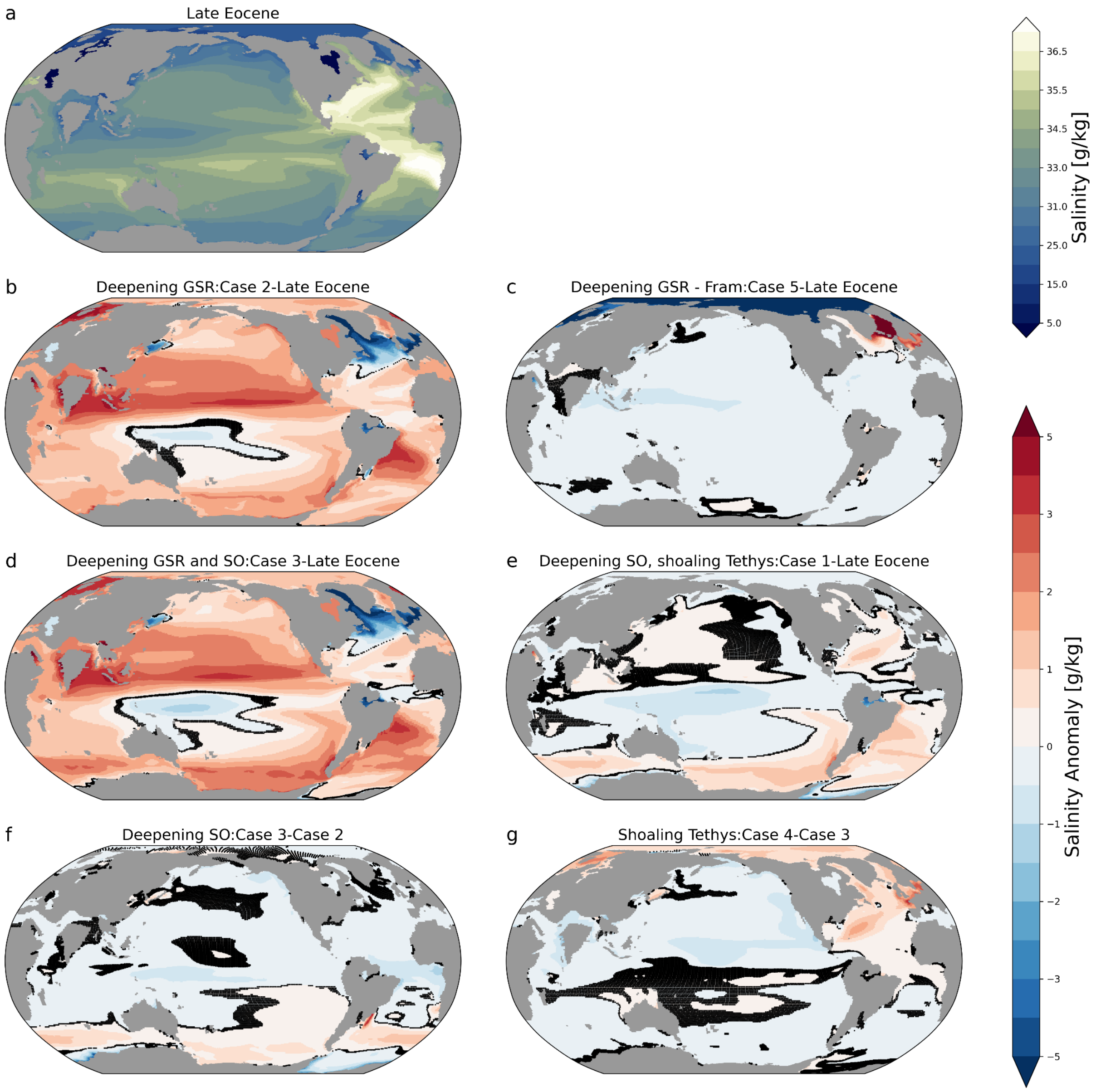

Figure S6: Sea Surface Salinity (SSS) using period 1450-1500. a) mean SSS for the Late Eocene run. SSS anomalies from b) deepening the GSR c) deepening GSR versus Fram Strait d) deepening the GSR and the SO gateways e) Deepening the SO gateways and shoaling the Tethys and f) deepening the SO gateways alone g) shoaling the Tethys alone. Black dots cover regions where the means are not significantly different from each other at 95% confidence level (2-sided student's t-test).

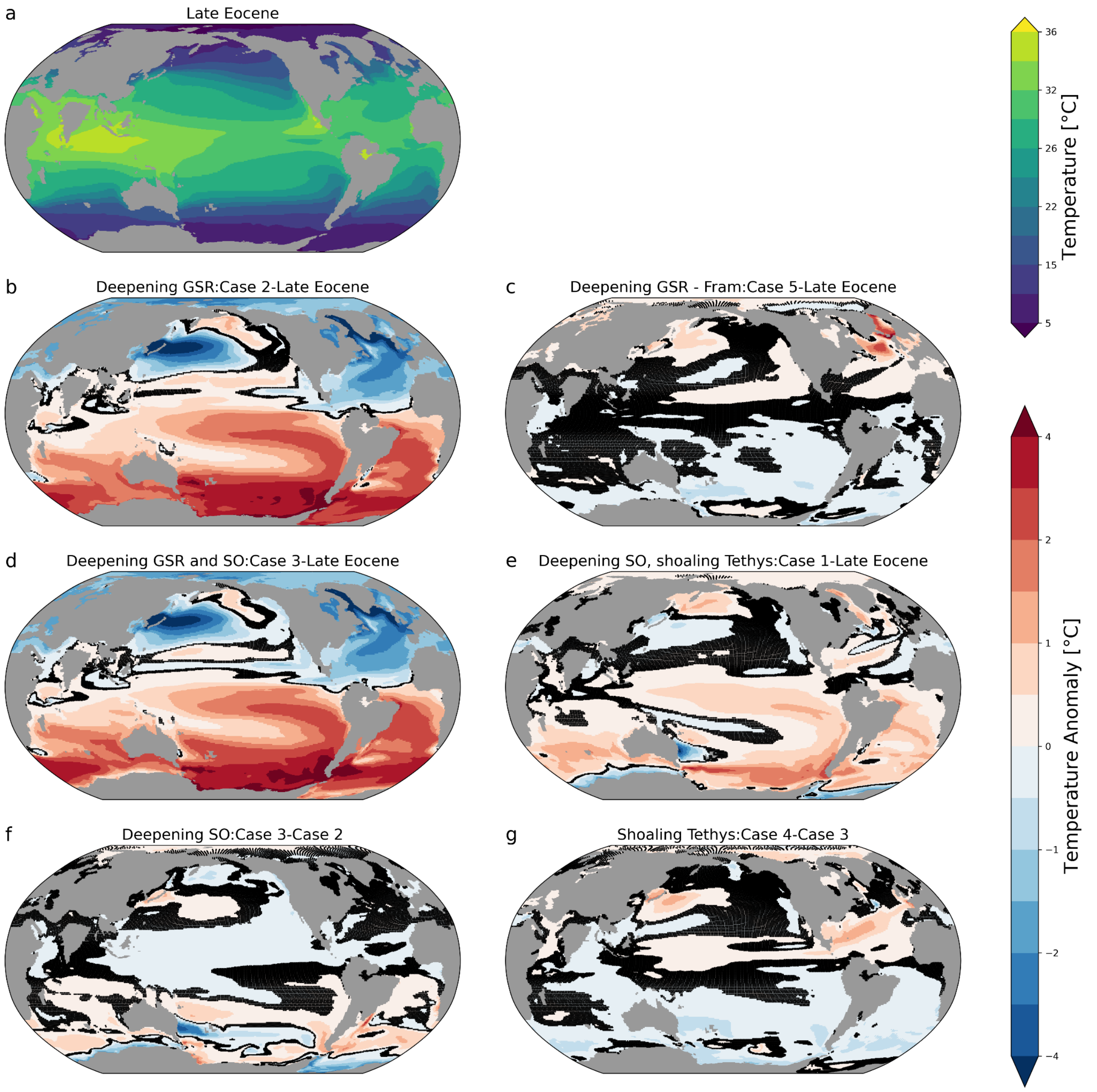

Figure S7: Sea Surface Temperatures (SSTs) using period 1450-1500. a) mean SST for the Late Eocene run. SST anomalies from b) deepening the GSR c) deepening GSR versus Fram Strait d) deepening the GSR and the SO gateways e) Deepening the SO gateways and shoaling the Tethys and f) deepening the SO gateways alone g) shoaling the Tethys alone. Black dots cover regions where the means are not significantly different from each other at 95% confidence level (2-sided student's t-test).

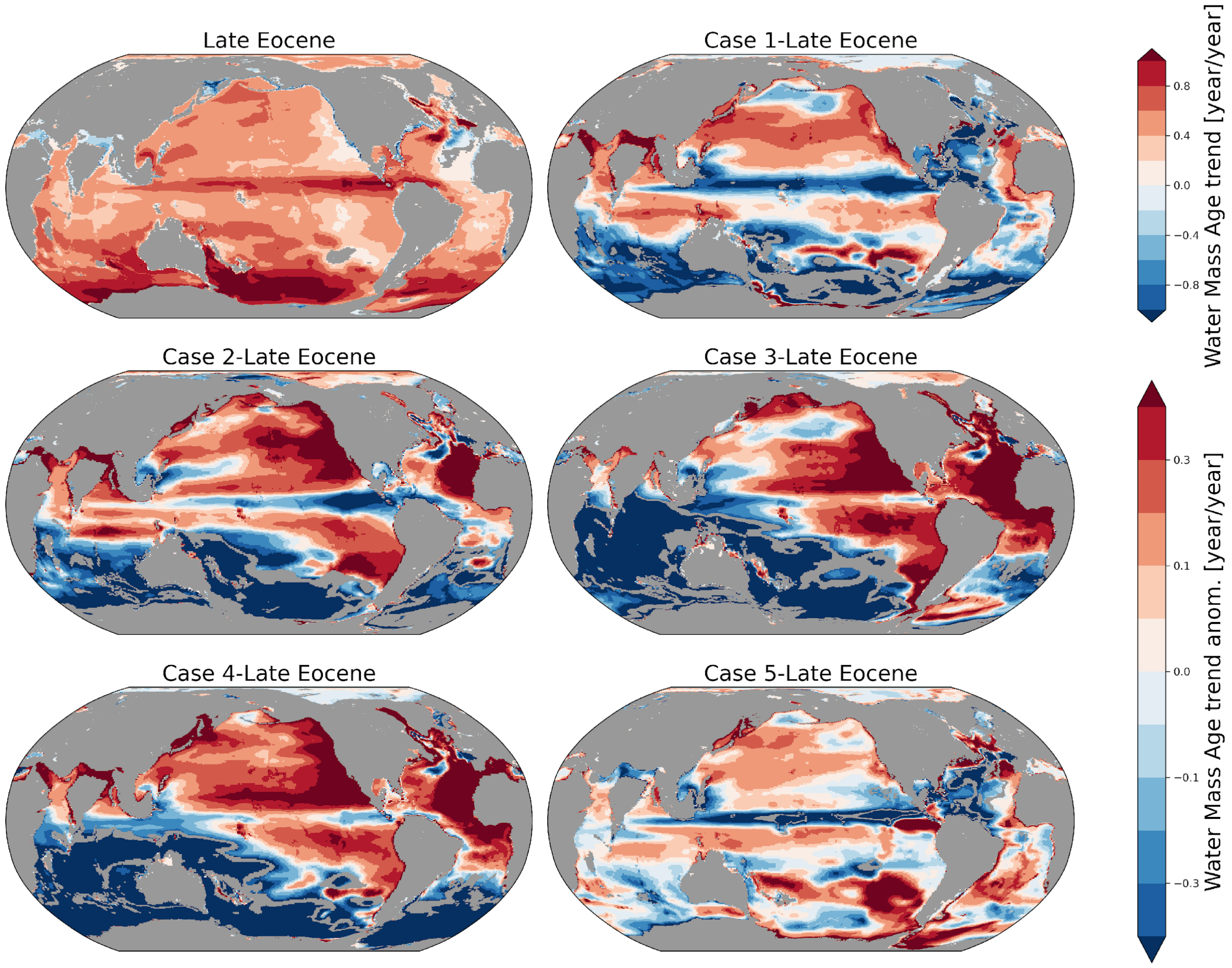

Figure S8: Ventilation measured as trend in the vertical mean water mass age. Panel (a) shows the trend in the Late Eocene climate whereas panels (b)-(f) show the anomaly when compared to the Late Eocene climate. The trend is based on the ideal age tracer, that is set to 0 at the surface and otherwise ages at the same rate as the simulations - thus representing the time since a water parcel was last in contact with the atmosphere. The trends are calculated by linear least squares fit to the years 1450-1500 of each case and the global average trend is quoted in the panel titles. Trends that are not significant at 5% level are stippled with grey dots.

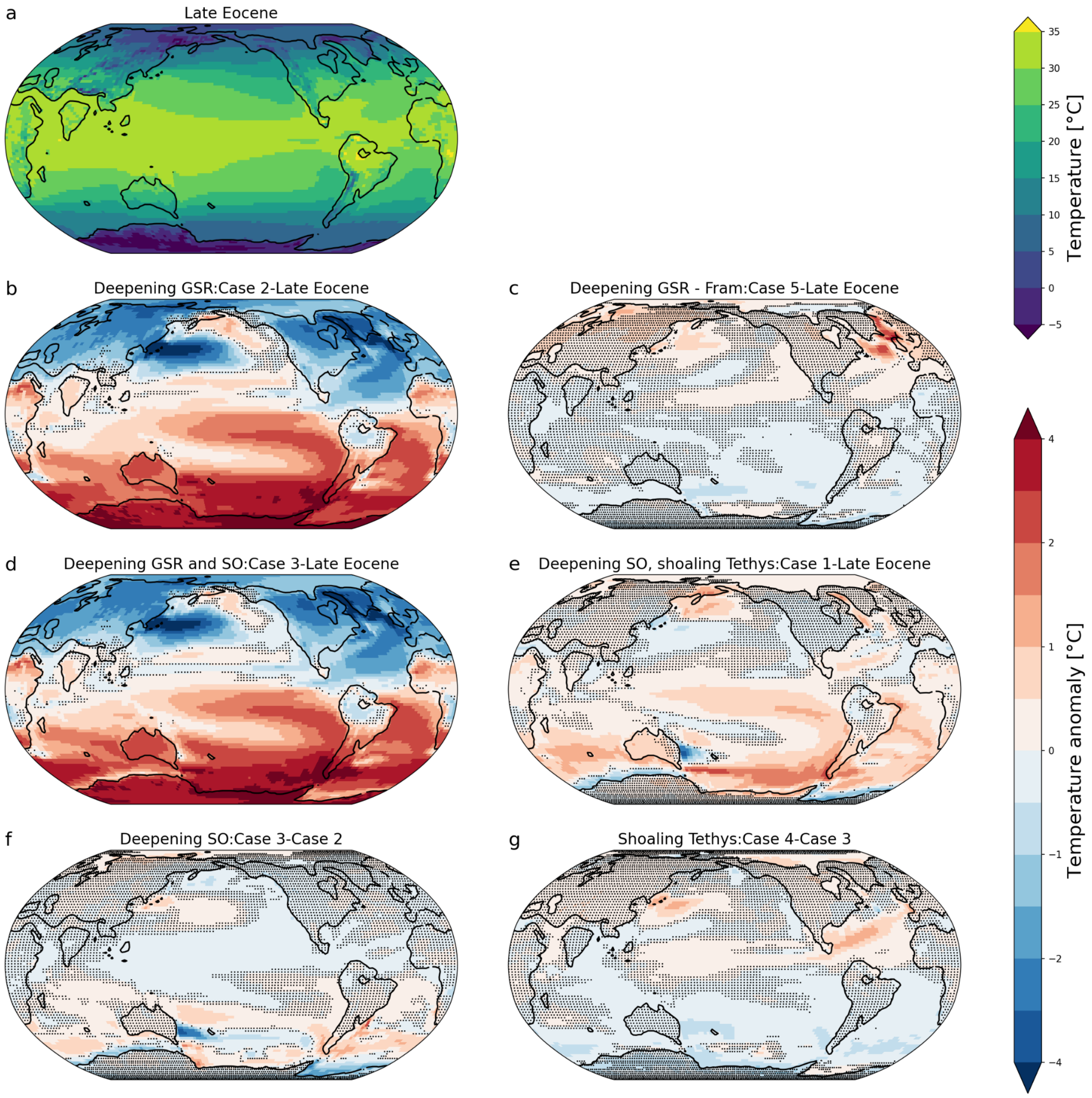

Figure S9: Surface Air Temperatures (SAT) using period 1450-1500. a) mean SAT for the Late Eocene run. SAT anomalies from b) deepening the GSR c) deepening GSR versus Fram Strait d) deepening the GSR and the SO gateways e) Deepening the SO gateways and shoaling the Tethys and f) deepening the SO gateways alone g) shoaling the Tethys alone. Gray dots cover regions where the means are not significantly different from each other at 95% confidence level (2-sided student's t-test).

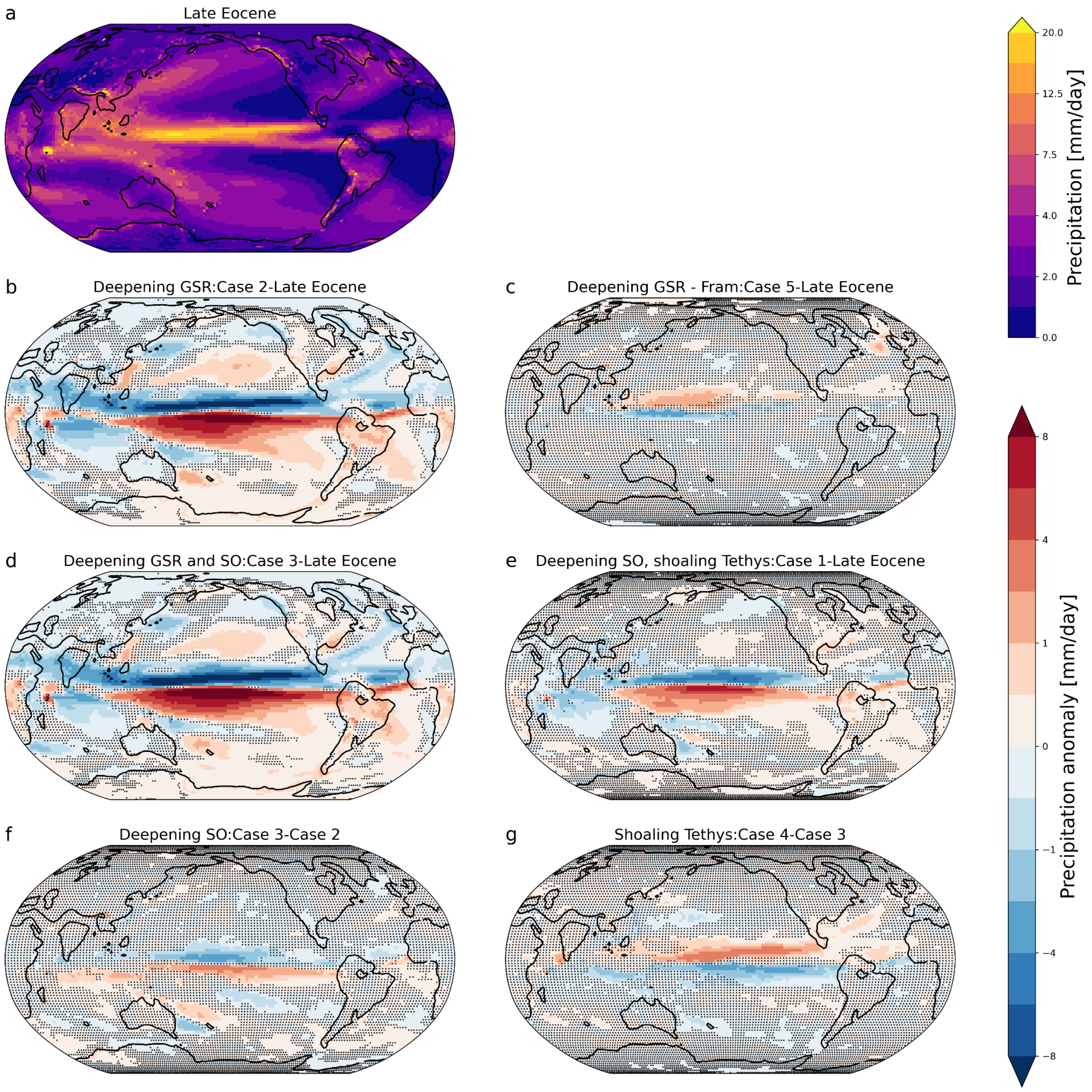

Figure S10: Annual precipitation using period 1450-1500. a) mean annual precipitation for the Late Eocene run. Annual precipitation anomalies from b) deepening the GSR c) deepening GSR versus Fram Strait d) deepening the GSR and the SO gateways e) Deepening the SO gateways and shoaling the Tethys and f) deepening the SO gateways alone g) shoaling the Tethys alone. Gray dots cover regions where the means are not significantly different from each other at 95% confidence level (2-sided student's t-test).

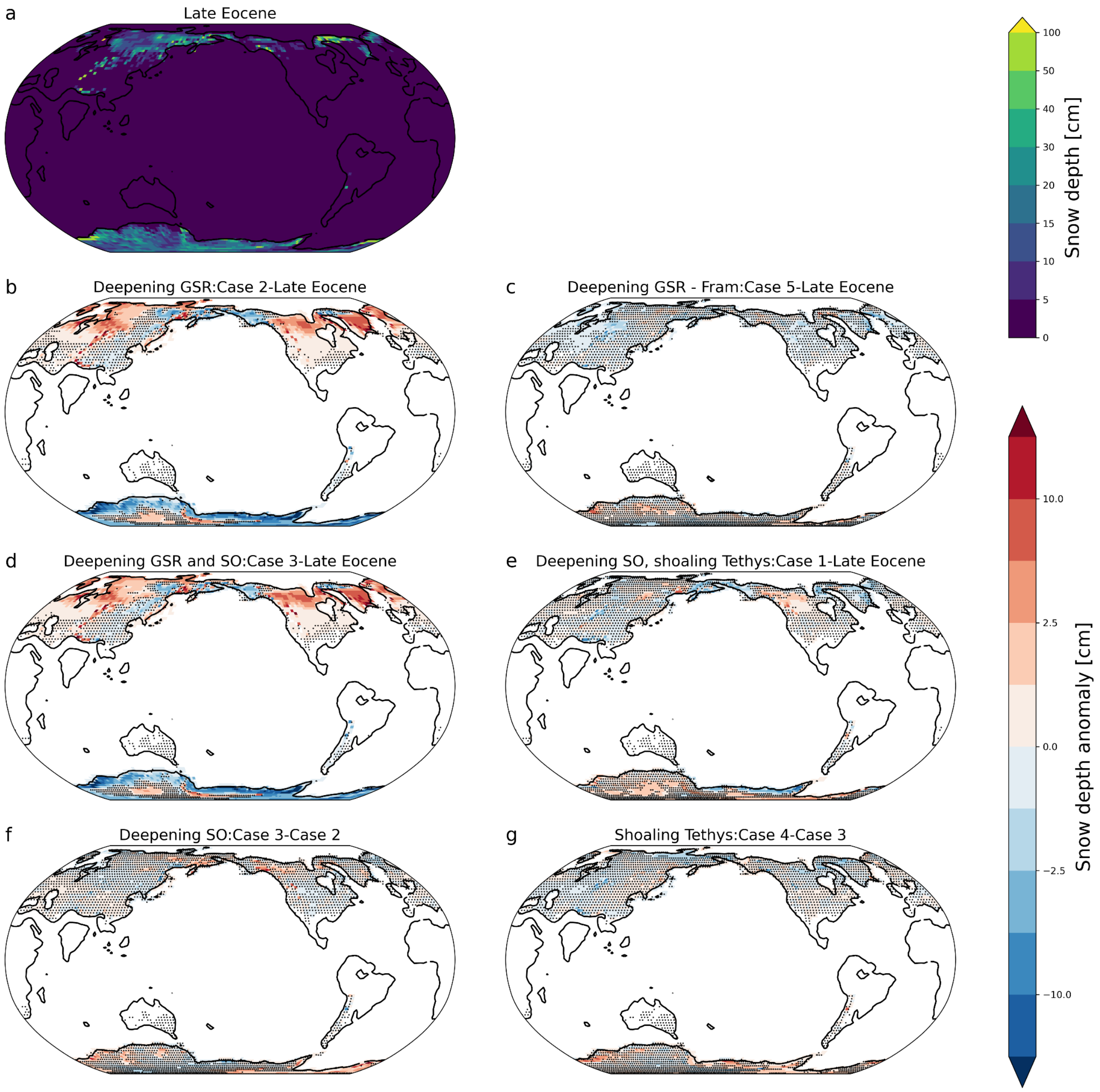

Figure S11: Winter snow depth (June-July-August for the Southern Hemisphere and December-January-February for the Northern Hemisphere) using years 1450-1500. a) mean winter snow depth for the Late Eocene run. Winter snow depth anomalies from b) deepening the GSR c) deepening GSR with closed Fram Strait d) deepening the GSR and the SO gateways e) Deepening the SO gateways and shoaling the Tethys and f) deepening the SO gateways alone g) shoaling the Tethys alone. Gray dots cover regions where the means are not significantly different from each other at 95% confidence level (2-sided student's t-test).

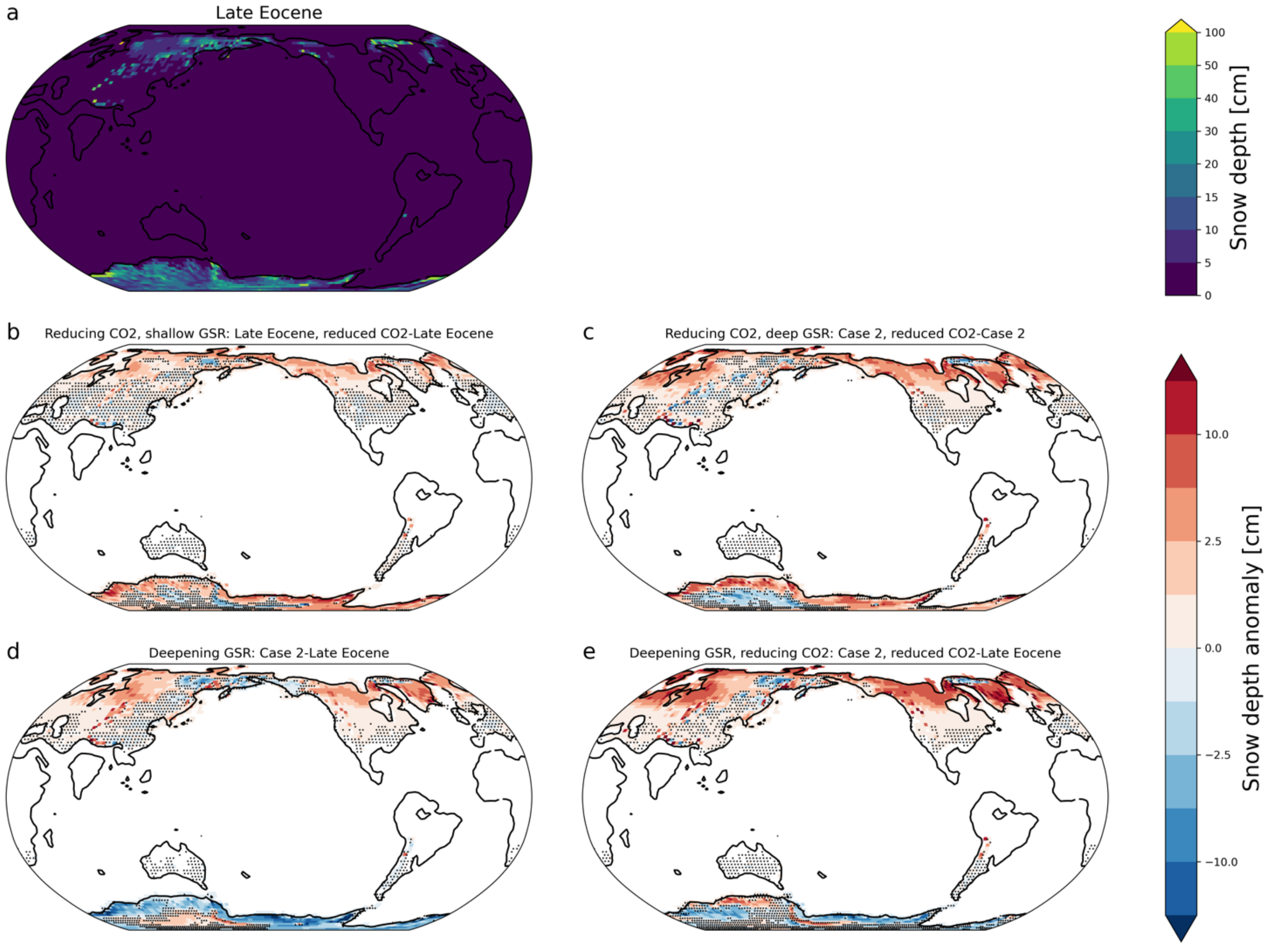

Figure S12: Winter snow depth (June-July-August for the Southern Hemisphere and December-January-February for the Northern Hemisphere) using years 1450-1500. a) mean winter snow depth for the Late Eocene run. Winter snow depth anomalies from b) reducing the CO_2 by 33 % for the late Eocene case. c) reducing the CO_2 by 33 % for case 2 d) Deepening the GSR and e) deepening the GSR and reducing CO_2. Gray dots cover regions where the means are not significantly different from each other at 95% confidence level (2-sided student's t-test).

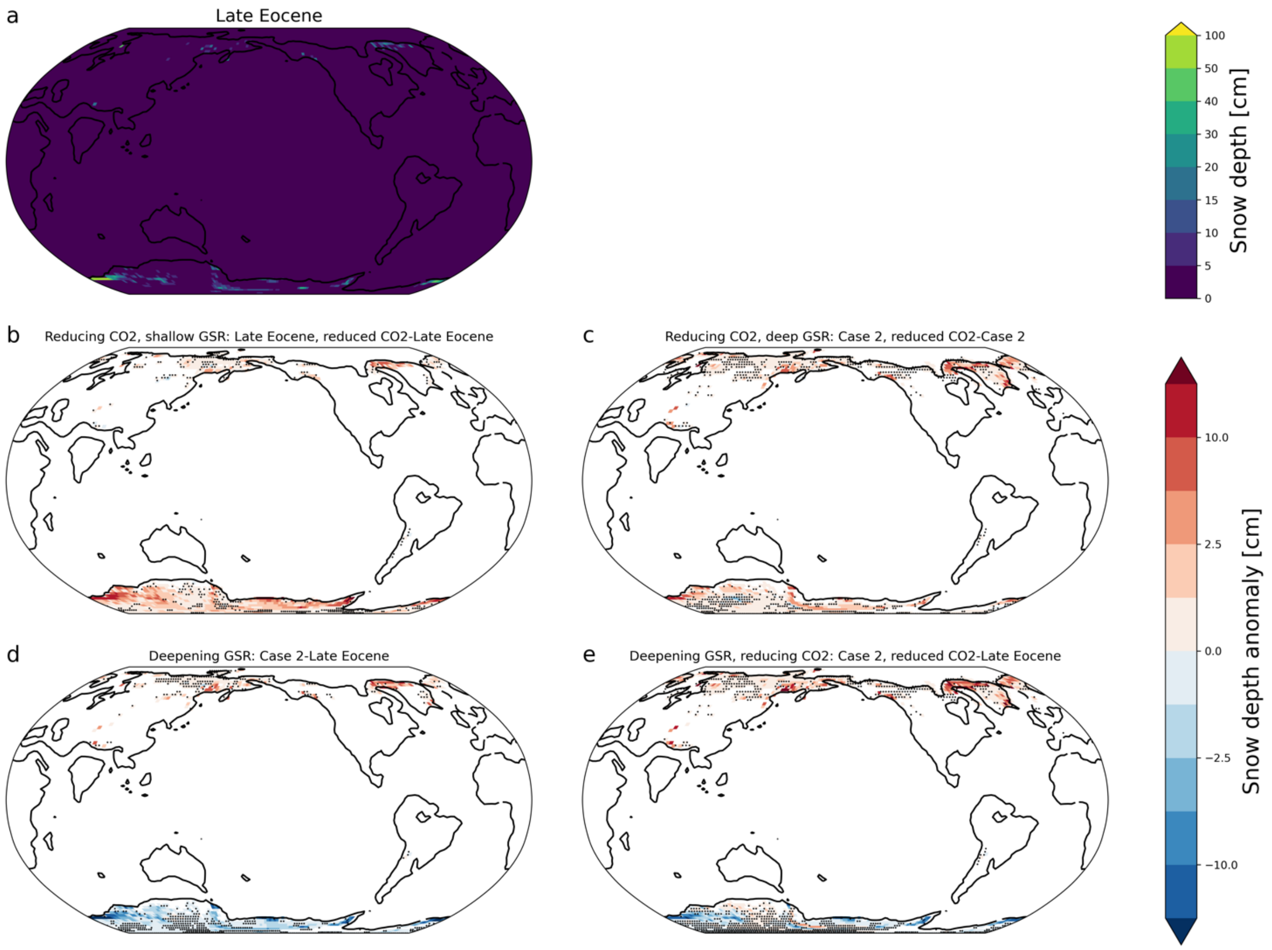

Figure S13: Summer snow depth (December-January-February for the Southern Hemisphere and June-July-August for the Northern Hemisphere) using years 1450-1500. a) mean winter snow depth for the Late Eocene run. Winter snow depth anomalies from b) reducing the CO_2 by 33 % for the late Eocene case. c) reducing the CO_2 by 33 % for case 2 d) Deepening the GSR and e) deepening the GSR and reducing CO_2. Gray dots cover regions where the means are not significantly different from each other at 95% confidence level (2-sided student's t-test).

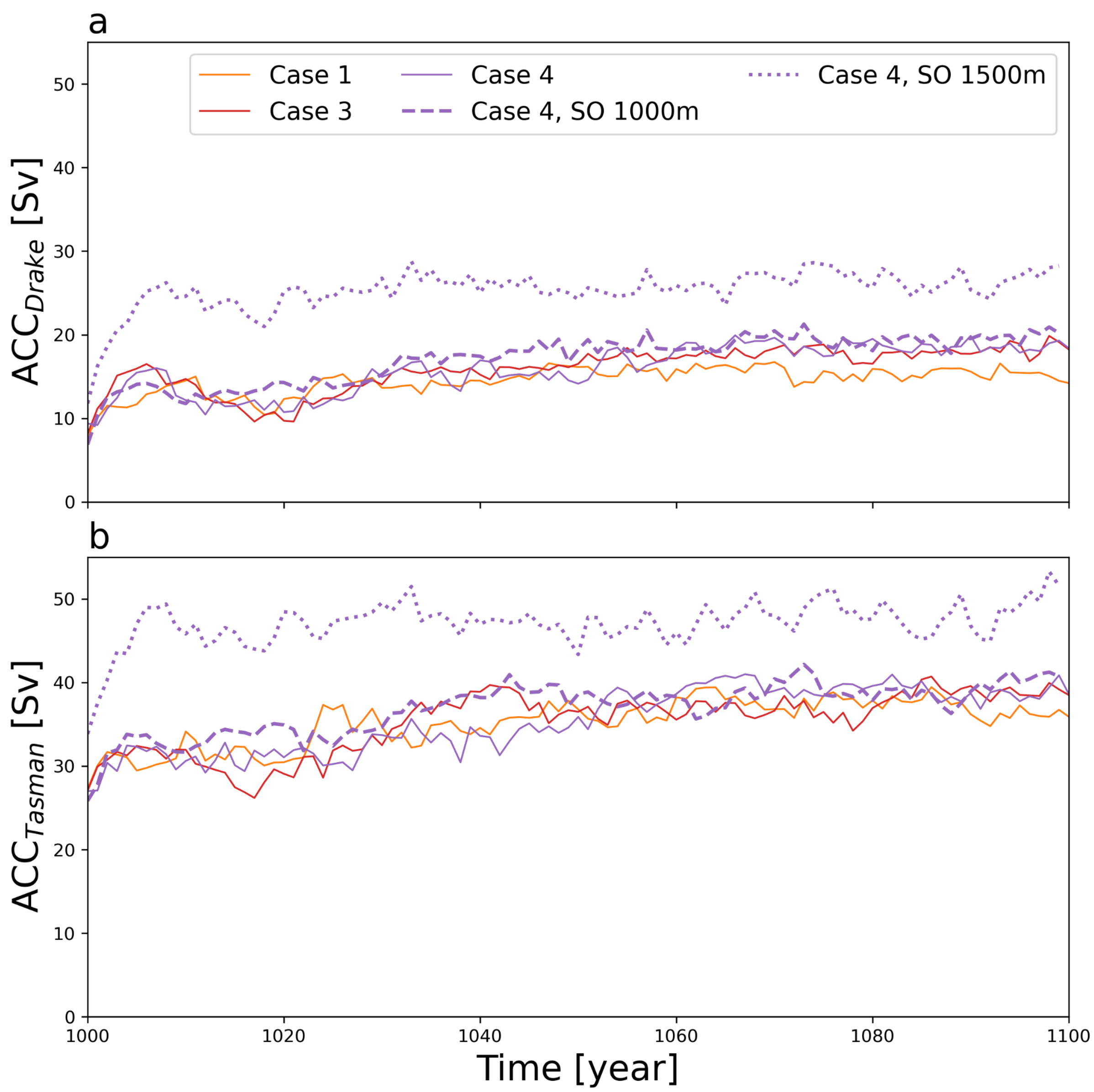

Figure S14: Southern Ocean circulation metrics when testing further deepening of the Southern Ocean (SO) gateways beyond realistic values for the EOT. a) Transport through the Drake Passage. b) Transport through the Tasman Gateway. 'Case 4, SO 1000m' and 'Case4, SO 1500m' are simulations where the Tasman gateway and Drake Passage are deepened beyond realistic depths at 34 Ma (to 1000 m and 1500 m, respectively), cases 1 and 3 are included here as SO gateways are open in these simulations as well.

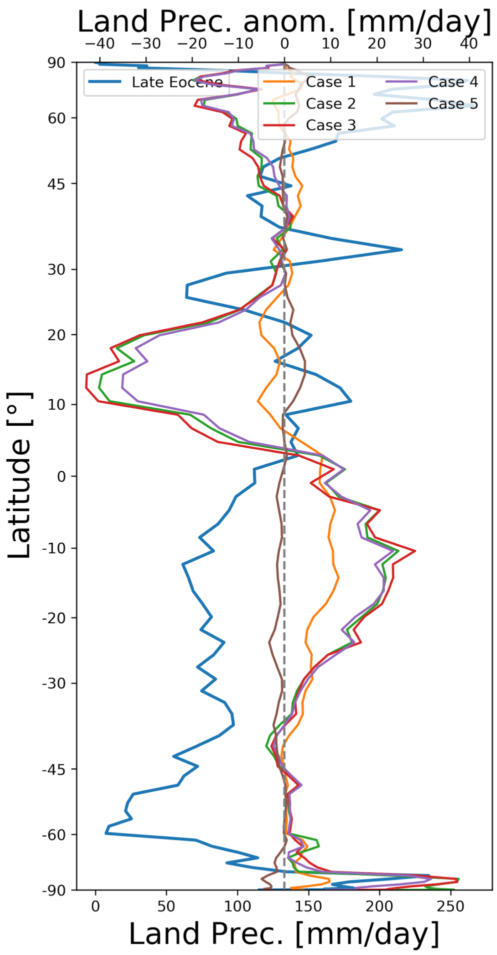

Figure S15: Annual mean zonal mean precipitation over land for the Late Eocene case (scale at the bottom) and anomalies for the other cases (scale at the top) in respect to the Late Eocene case. Note that when the AMOC collapses, as in Cases 2-4, there is a clear shift in precipitation between the Northern and Southern Hemispheres.

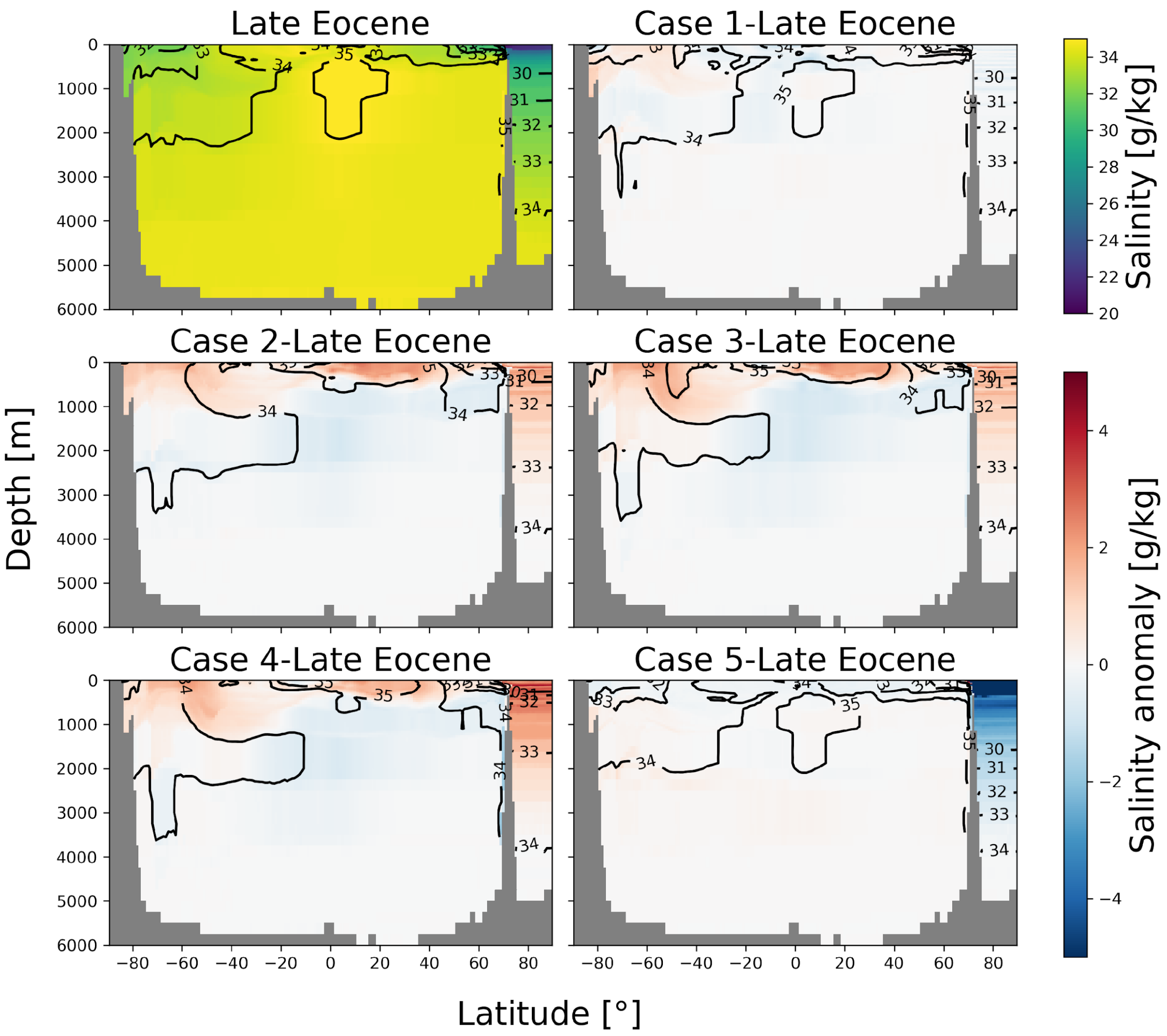

Figure S16: Change in global zonal mean salinity as a function of depth and latitude.

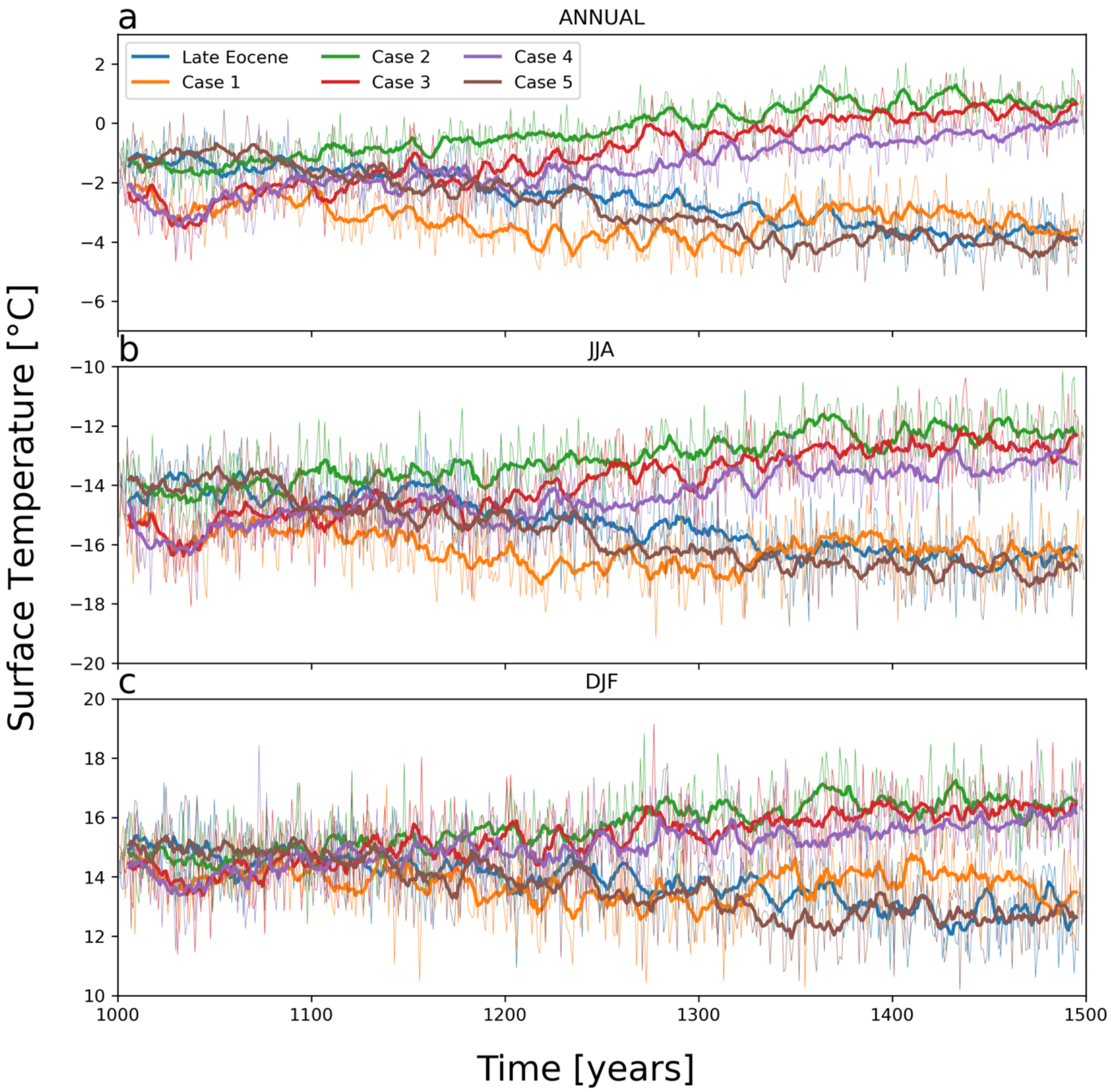

Figure S17: Antarctic land surface temperature for different seasons a) annual b) winter (JJA) c) summer (DJF). Thick lines show a 10-year running averages whereas thin lines show annual values.
